# Engineering a Carbohydrate-processing Transglycosidase into Glycosyltransferase for Natural Product Glycodiversification

**DOI:** 10.1038/srep21051

**Published:** 2016-02-12

**Authors:** Chaoning Liang, Yi Zhang, Yan Jia, Wenzhao Wang, Youhai Li, Shikun Lu, Jian-Ming Jin, Shuang-Yan Tang

**Affiliations:** 1 CAS Key Laboratory of Microbial Physiological and Metabolic Engineering, Institute of Microbiology, Chinese Academy of Sciences, Beijing 100101, China; 2 Beijing Key Laboratory of Plant Resources Research and Development, Beijing Technology and Business University, Beijing 100048, China; 3 School of Chemistry and Biotechnology, Yunnan Minzu University, Kunming, China; 4 State Key Laboratory of Mycology, Institute of Microbiology, Chinese Academy of Sciences, Beijing, China; 5 University of Chinese Academy of Sciences, Beijing, China

## Abstract

Glycodiversification broadens the scope of natural product-derived drug discovery. The acceptor substrate promiscuity of glucosyltransferase-D (GTF-D), a carbohydrate-processing enzyme from *Streptococcus mutans*, was expanded by protein engineering. Mutants in a site-saturation mutagenesis library were screened on the fluorescent substrate 4-methylumbelliferone to identify derivatives with improved transglycosylation efficiency. In comparison to the wild-type GTF-D enzyme, mutant M4 exhibited increased transglycosylation capabilities on flavonoid substrates including catechin, genistein, daidzein and silybin, using the glucosyl donor sucrose. This study demonstrated the feasibility of developing natural product glycosyltransferases by engineering transglycosidases that use donor substrates cheaper than NDP-sugars, and gave rise to a series of α-glucosylated natural products that are novel to the natural product reservoir. The solubility of the α-glucoside of genistein and the anti-oxidant capability of the α-glucoside of catechin were also studied.

As an emerging field of interest in modern biotechnology, glycodiversification of natural products has broadened the scope of drug discovery^1^,^2^,^3^,^4^,^5^,^6^,^7^,^8^. Carbohydrate residues have various effects on drug properties including pharmacokinetics, pharmacodynamics, solubility, and membrane transport. Enzyme-based natural product glycodiversification provides stereo- and regio-selectivity and has seen great progress in recent years.

The major biocatalysts of natural product glycosylation in nature are glycosyltransferases (GTs), which require sugar nucleotide (NDP-sugar) glycosyl donors or other activated glycosyl donor substrates. A wealth of natural-product glycosides including hormones and antibiotics are naturally biosynthesized through GTs. Recent research has aimed to improve GT catalytic efficiency or reduce reaction costs^9^,^10^,^11^,^12^. For example, the promiscuous substrate specificity of OleD, an oleandomycin GT from *Streptomyces antibioticus*, was expanded through directed evolution. OleD mutant derivatives transformed the glycosyl moiety to an expanded spectrum of natural products, and made use of various NDP-sugar glycosyl donors^4^,^5^. Studies of four glycosyltransferases from two distinct natural product biosynthetic pathways (calicheamicin and vancomycin) suggest these GTs readily catalyze reversible reactions, allowing sugars and aglycons to be exchanged with ease, they are therefore useful for generating exotic nucleotide sugars and enhancing natural product diversity^1^. The catalytic reversibility of other GTs has also been identified^11^,^12^,^13^. YjiC from *Bacillus licheniformis* has been used to modify commercially available isoflavonoids in an engineered *Escherichia coli* strain^14^. It was also found that by domain swapping, the substrate specificity of GTs could be broadened^15^,^16^,^17^.

Glycoside hydrolases act as carbohydrate-processing enzymes in nature. Apart from hydrolysis activities, some of them were reported to exhibit high transglycosylation activities, also called transglycosidase. The transglycosylation activity of glycoside hydrolases has been applied in synthesizing oligosaccharides as well as UDP-activated oligosaccharides using starch or sugars as the donor substrates^18^,^19^,^20^,^21^. Glycosynthases, a family of mutant glycoside hydrolases deficient in hydrolysis activity, could synthesize glycans with high yield, however, the activated donor substrates, glycosyl fluorides, were required^22^. Some transglycosidases have been reported to produce some natural product glycosides in low amount^23^,^24^,^25^,^26^. Usually these transglycosidases catalyze the transfer of donor glycosides to the carbohydrate moiety of a natural product glycoside rather than to the aglycon, forming a multiple carbohydrate glycosylated natural product. Some transglycosidases can use simple small molecules as the acceptor substrates, such as hydroquinone and ascorbic acid^27^,^28^,^29^,^30^. By application of directed evolution methodologies, the activities of glycoside hydrolases or glycosynthases have been improved towards expanded sugar-donor or acceptor substrates^31^,^32^,^33^; however, the transglycosylation activities on non-glycosylated natural-product acceptors still remain low. To meet this end, recently, a microbial amylosucrase and a rice transglucosidase have been engineered for improved transglycosylation activity on luteolin and kaempferol, repectively^34^,^35^.

Among the transglycosidases, glucansucrases (EC 2.4.1.5) catalyze the hydrolysis of sucrose and transfer the glucosyl moiety to form a growing glucose polysaccharide through α-1,6/α-1,3 glycosidic bonds^36^,^37^,^38^ (Figure S1). Glucosyltransferase-D (GTF-D), a glucansucrase from the dental pathogen *Streptococcus mutans*, was reported able to mediate glycosylation of catechol, catechin, 4-methylcatechol, and 3-methoxycatechol via α-glucosidic bonds with low efficiency^39^,^40^ .

In this study, the acceptor substrate promiscuity of GTF-D was expanded by a protein engineering strategy that yielded a derivative with significantly improved capacity to glucosylate various flavonoid compounds using cheap and easily obtained sucrose as the glucosyl donor. The transglycosyl efficiency of GTF-D was also improved. By analyzing the structures of the glucoside products released by the GTF-D mutant, we revealed the glucosylation patterns and the regioselectivity of the glucosylation reaction. In addition, the mutant enzyme gave rise to a series of α-glucosylated flavonoids novel to the natural product reservoir. Glycosylation is also known to improve the solubility, stability or bioavailability of flavonoids^8^,^34^,^35^. The newly developed enzyme has great potential applications in natural products glycodiversification. The successful application of the protein engineering strategy in this study demonstrates its potential utility for engineering transglycosidases for natural product glycodiversification.

## Results

### Site-saturation mutagenesis library screening

**Figure 1:**
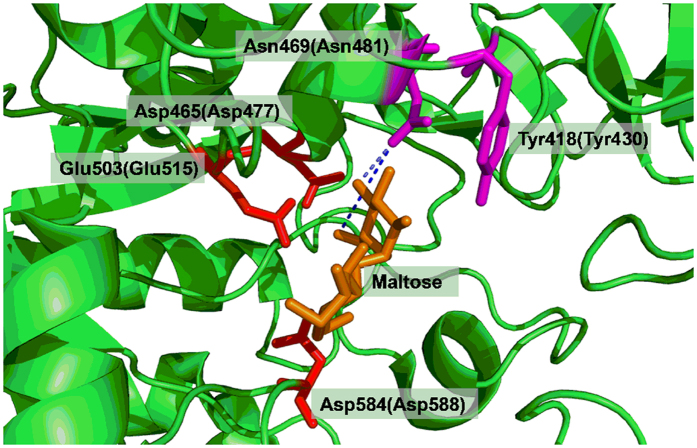
Structure of the GTF-SI-maltose complex (PDB ID: 3AIB, with sequence identity of 51% with GTF-D) 41. According to the sequence alignment, the catalytic amino acids for GTF-D were Asp584, Glu503 and Asp465. The selected amino acids for saturation mutagenesis were Tyr418 and Asn469. The corresponding numbering of the amino acids in GTF-SI sequence was indicated in parentheses.

**Figure 2:**
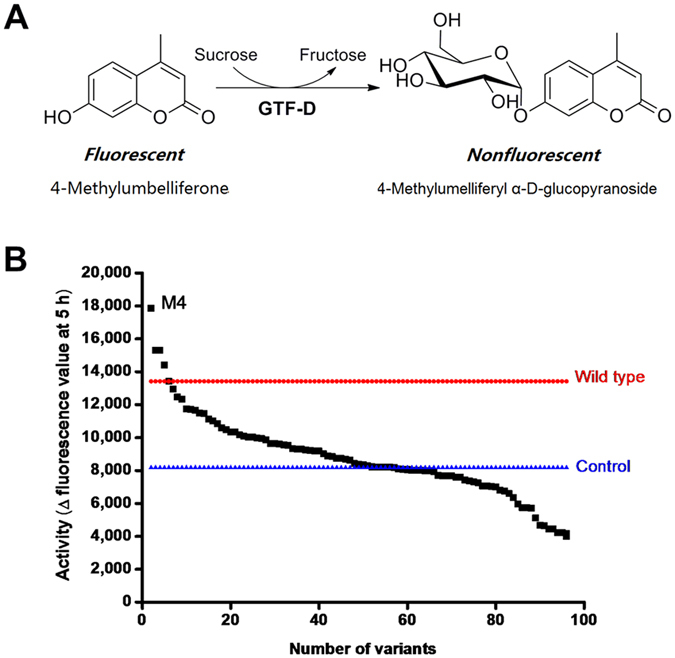
(**A**) Scheme of the glycosylation reaction based on which the high-throughput screening method was developed. (**B**) The representative activity data of glycosylation of fluorescent 4-MU illustrating ~100 random members from the GTF-D saturation mutagenesis library screening. The wild-type enzyme and mutant M4 were indicated. Strain BL21(DE3) harboring plasmid pET28a was used as control. Activities were calculated as the fluorescence differences of the variants between 0 (before the reaction) and 5 h (after the reaction).

GTF-D belongs to glycoside hydrolase family 70 (GH70). The sequence of GTF-D was aligned with those of glucansucrase GTF-SI from *S. mutans* (51% sequence identity with GTF-D)^41^ and GTF180 from *Lactobacillus reuteri* 180 (50% sequence identity with GTF-D)^42^. According to the crystal structure of GTF-SI-maltose complex (PDB ID 3AIB), Tyr 430 participated in hydrophobic interactions with the glucosyl moiety in subsite +1, while Asn481 forms hydrogen bonds with the C4 and C6 hydroxyl groups of the glucosyl moiety in subsite +1^38^. Thus these two residues are critical for the recognition of the moiety in subsite +1 or the incoming non-sugar acceptor substrate. In GTF-D, these two residues are conserved as Tyr418 and Asn469 within the acceptor substrate binding pocket, both of which were selected for a simultaneous site-saturation mutagenesis (Fig. 1). The semi-rational mutagenesis library was screened for quenching of coumarin 4-methylumbelliferone (4-MU) fluorescence by masking of the C-7 hydroxyl^4^, forming 4-methylumelliferyl α-d-glucopyranoside (4-MUG) (Fig. 2A). This screening method was previously used in engineering the glycosyltransferase OleD for expanded substrate promiscuity, with the donor substrate and product being UDP-glucose and 4-methylumbelliferyl β-d-glucopyranoside, respectively, instead^4^. Among the variants displaying the largest decrease of fluorescence intensity during the catalytic reaction (Fig. 2B), mutant M4 was selected from a total of 1,000 mutants. Sequencing of the gene of mutant M4 revealed Y418R and N469C amino acid substitutions.

### Glycosylation capacity of mutant M4 towards various acceptor substrates

**Figure 3:**
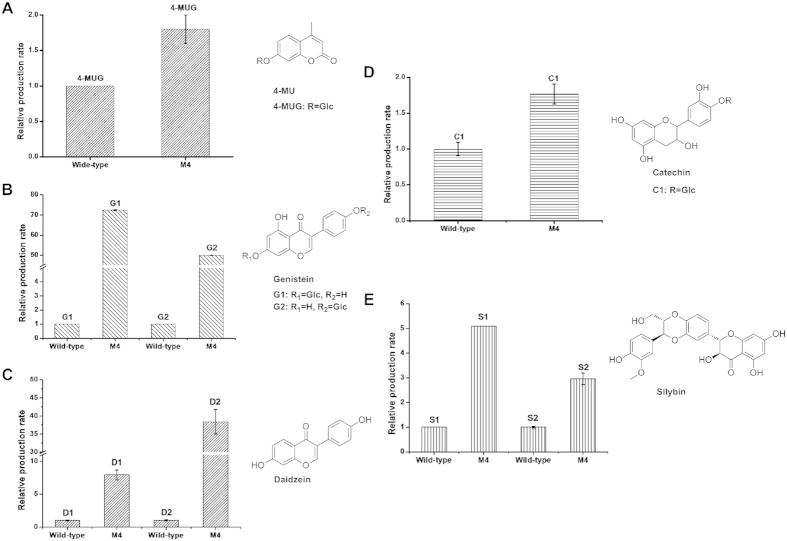
Relative production rates of the transglycosylation products obtained from 4-MU (**A**), genistein (**B**), daidzein (**C**), catechin (**D**) and silybin (**E**) catalyzed by wild-type GTF-D and its M4 mutant. The relative production rate was calculated as the fold of production by mutant M4 relative to the production by wild-type enzyme (set as 1) for each product. All reported data were the mean of three independent data points. The error bars represent standard deviations.

**Figure 4:**
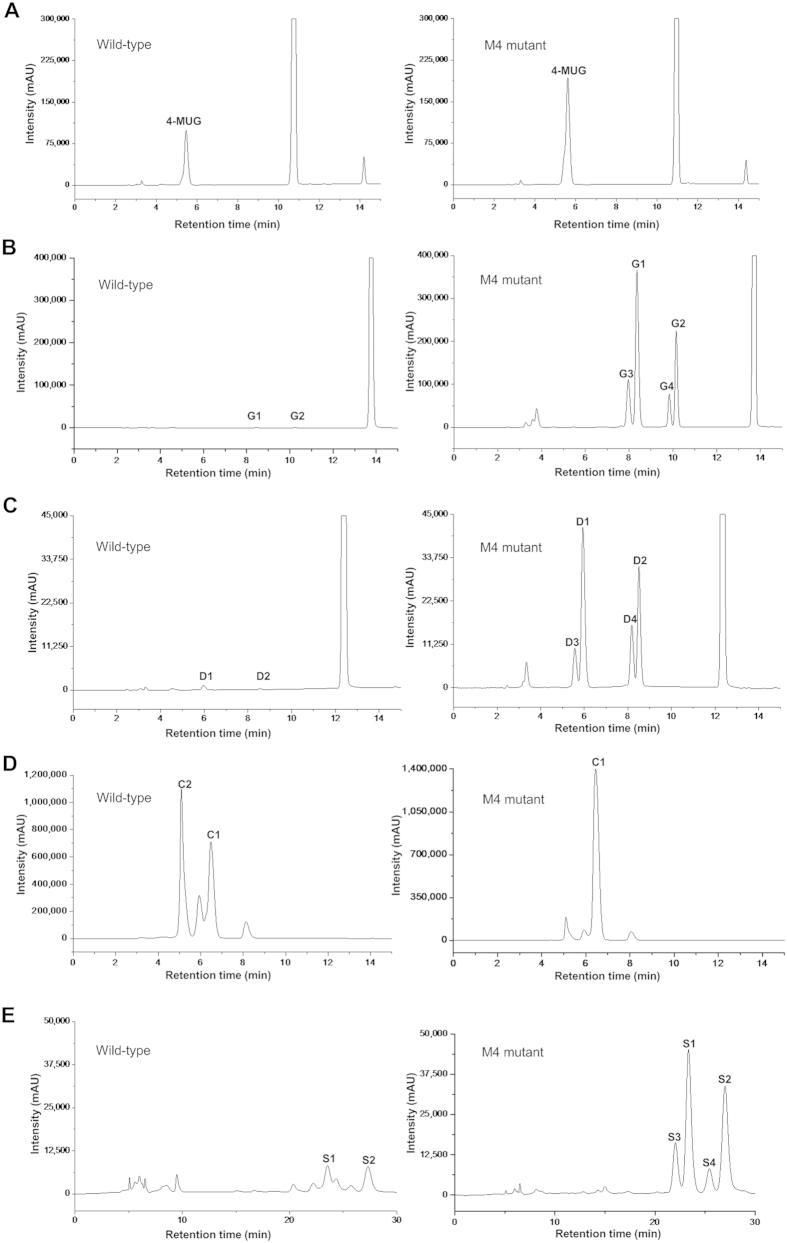
HPLC chromatography of the glucoside products from 4-MU (**A**), genistein (**B**), daidzein (**C**), catechin (**D**) and silybin (**E**) catalyzed by wild-type GTF-D and its M4 mutant. G1, G2, D1, D2, S1 and S2 were monoglucosylated products, while G3, G4, D3, D4, S3 and S4 were diglucosylated products, as revealed by LC-MS analyses.

HPLC analysis showed that mutant M4 conferred 1.8-fold higher production of the transglycosylation product than the wild-type enzyme on the screening substrate 4-MU (Figs 3A and 4A). The transglycosylation capacity of this mutant enzyme on flavonoid compounds which share similar structure properties with 4-MU (catechin, daidzein, genistein, silybin) were also assessed. As predicted, mutant M4 exhibited significantly improved transglycosylation activities towards these flavonoid acceptors (Figs 3,4 and Table 1), among which the transglycosylation activity of wild-type GTF-D on genistein and daidzein were almost non-detectable (Fig. 4). As reported previously^40^, the wild-type GTF-D showed transglycosylation activity on catechin and two products, C1 and C2, were formed. C1 was a monoglucosylated product and C2 was a diglucosylated product. Mutant M4 was found to mainly produce C1 whose production exhibited an 1.8-fold of increase as compared with the wild-type enzyme.

The kinetic parameters of wild-type and the mutant GTF-D were compared on the flavonoid substrates catechin and genistein individually (Table 2). For the acceptor substrate catechin, a 1.7-fold of increase in *k*_cat_/*K*_m_ was observed for mutant M4 as compared with the wild-type enzyme. Notably, on the transglycosylation of genistein, the catalytic efficiency of mutant M4 was significantly higher than that of wild-type GTF-D, whose transglycosylation products were hardly detected.

### Glycosylation pattern analysis

The glucosylated products were analyzed by LC-MS and NMR. Four glucosylated products of genistein were identified (Fig. 4B), among which two major products were monoglucosylated (G1 and G2) and two minor products were diglucosylated (G3 and G4). Varying the concentrations of sucrose, the donor substrate, would not influence the overall product distributions very much (Figure S2).

To elucidate the structures of the two major reaction products of genistein, G1 and G2 were purified from the reaction products. The molecular formula of G1 was defined as C_21_H_20_O_10_ by ^13^C NMR data and its positive ion HR-ESI-MS (*m*/*z* 433.1129 [M + H]^+^. calcd for C_21_H_21_O_10_, 433.1129). In ^1^H spectrum of G1, an anomeric proton signal was identified at δ_H_ 6.29 (1H, d, *J* = 2.4 Hz). The *J* value (<6 Hz) of the anomer of the sugar moiety indicated the α-orientation at the anomeric center of the d-glucopyranosyl unit. The ^13^C NMR data of G1 (Table S2) were in good consistent with those of genistin^43^. In the HMBC spectrum of G1, the anomeric proton signal of glucopyranosyl unit at δ_H_ 6.29 correlated with δ_C_ 164.2, indicating that the glucopyranosyl unit was attached to the hydroxyl of the aglycone C-7. On the basis of the above evidence, G1 was identified as genistein-7-*O-*α-d-glucopyranoside (Figures S3 and S4).

The molecular formula of G2 was defined as C_21_H_20_O_10_ by positive ion HR-ESI-MS (*m*/*z* 433.1131 [M + H]^+^. calcd for C_21_H_21_O_10_, 433.1129). The proton and carbon signals of G2 were assigned by analysis of 1D and 2D NMR (Table S2). In ^1^H spectrum of G2, *J* = 3.2 Hz (<6 Hz) of the anomeric proton signal at δ_H_ 6.13 indicated α-orientation of at the anomeric center of d-glucopyranosyl unit. Correlation of δ_H_ 6.13 (H-Glc-1) with δ_C_ 158.9 (C-4′ of aglycone) was observed in the HMBC spectrum of G2. Thus, G2 was identified as genistein-4′-*O-*α-d-glucopyranoside (Figures S3, S4).

Mutant M4 therefore exhibited remarkably improved transglycosylation activity on genistein as compared with wild-type GTF-D, with a transglycosylation bias on the C-7 and C-4′ hydroxyl groups. G1 and G2 have not been reported before and were novel to the natural product reservoir.

The glycosylation pattern of mutant M4 on daidzein was similar to that of genistein. Two major products (D1 and D2) and two minor products (D3 and D4) were observed by HPLC (Fig. 4C). LC-MS revealed that two major products D1 and D2 had the molecular formula of C_21_H_20_O_9_ (D1: *m*/*z* 417.1265 [M + H]^+^; D2: *m*/*z* 417.1277 [M + H]^+^), suggesting that D1 and D2 are daidzein monoglucosides, and two minor products D3 and D4 were defined as daidzein diglucosides based on their positive ion at *m*/*z* 579.1848 [M + H]^+^ and 579.1836 [M + H]^+^.

In the reaction catalyzed by mutant M4 on catechin, the main product produced was found to be monoglucosylated (catechin-4′-*O-*α-d-glucopyranoside, C1, Figure S3) as revealed by NMR, MS and HPLC (Fig. 4D, Table S2). The NMR data were identical to those reported previously^40^. Thus in the transglycosylation of catechin, mutant M4 displayed a transglycosylation bias on the C-4′ hydroxyl group.

Silybin was also tested as an acceptor substrate. Mutant M4 exhibited transglycosylation capability on silybin with glucoside products identified as two major monoglucosylated products (S1: *m*/*z* 645.1973 [M + H]^+^; S2: *m*/*z* 645.1965 [M + H]^+^) and two minor diglucosylated products (S3: *m*/*z* 807.2557 [M + H]^+^; S4: *m*/*z* 807.2542 [M + H]^+^) by LC-MS (Fig. 4E).

From the above results, the major transglycosylation products produced by GTF-D mutant M4 on the flavonoid substrates were monoglucosylated products. The C-7 or C-4′ hydroxyl were the preferred sites for transglycosylation.

### Solubility of the glucosylated product of genistein

**Figure 5:**
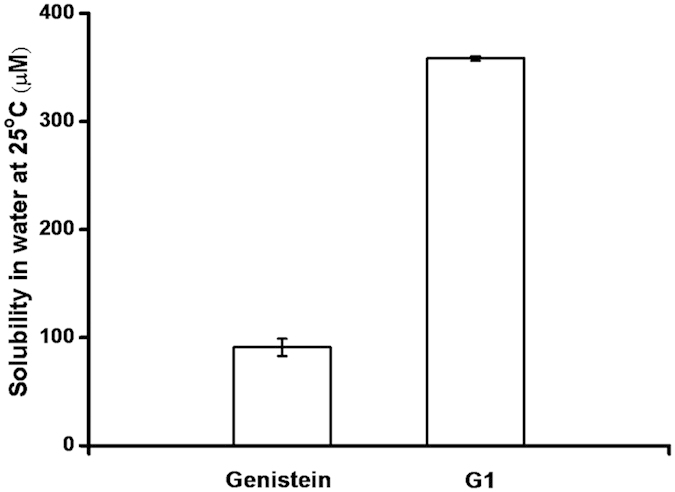
Solubility of genistein and genistein-7-*O-*α-d-glucopyranoside (G1) in water at 25 °C.

Glycosylation improves the solubility of otherwise poorly water-soluble natural products and improves their bioavailability. We compared the water solubility of genistein-7-*O-*α-d-glucopyranoside and genistein. It was found that genistein-7-*O-*α-d-glucopyranoside displayed an almost 4-fold increase in solubility (358 μM) at 25 ^o^C, compared with genistein (90 μM) (Fig. 5).

### Anti-oxidant activity of the glucosylated products

The glucose moieties of the transglycosylation products produced with the GTF-D mutant enzyme were all α-configured, whereas the glucose moieties of flavonoids existing in nature were exclusively β-configured. Therefore, the transglycosylation products obtained here represent a group of new compounds whose bioactivities are unknown. The anti-oxidant activities of catechin and catechin-4′-*O-*α-d-glucopyranoside were compared. With both methods, the anti-oxidant activities of the glucosylated form were not lower than those of the non-glucosylated form (Table 3).

### Docking study

The model structures of wild-type GTF-D and mutant M4 were generated based on the high-resolution crystal structure of *L. reuteri* 180 glucansucrase GTF180 (PDB ID 3HZ3)^42^. 4-MU was docked into the acceptor binding pocket of the two models individually. As shown in Figure S7, hydrogen bond between 4-MU and Arg418, Asp468 or Asn413 was observed in mutant M4. By contrast, these hydrogen bonds were not observed in the wild-type enzyme structure. Therefore, the three additional hydrogen bonds formed due to the two mutations may pull 4-MU close to the active center and contribute to the increased catalytic efficiency of the mutant enzyme on 4-MU as an acceptor substrate. The N413A mutant of M4 enzyme was constructed and found to display ~60% activity on substrate 4-MU as compared with the M4 enzyme.

## Discussion

Flavonoids are polyphenolic natural products that appear throughout the plant kingdom. They are frequently used in food, cosmetic and pharmaceuticals. Studies have demonstrated that flavonoid compounds generally exhibit anti-inflammatory, anti-oxidant and anti-tumor activities, mainly due to their polyphenol structures that protect against cardiovascular and coronary heart diseases or certain forms of cancer^34^,^44^,^45^,^46^,^47^. However, the major drawback of flavonoid compounds is their poor water solubility, which severely limits their application. Flavonoid glucosides that have mono- or oligoglucoside residues linked to the aglycons are usually much more soluble. Glucosides of catechin, for example, are 100-fold more soluble than catechin, and have significantly improved bioavailability^48^, thus demonstrating the importance of natural product glycosylation.

Although some transglycosidases catalyze the glycosylation of some small natural products, as natural carbohydrate processing enzymes, their recognition of drug-related natural products as the acceptor substrates is rather limited, which hampers their application in glycodiversification efforts. Glucansucrases hydrolyze sucrose and transfer the glucosyl moiety from sucrose to form glucans. In this study, we applied a protein engineering strategy to expand the substrate promiscuity of glucansucrase GTF-D, and enable it to transfer glucosyl moieties to various non-glycosylated flavonoids using sucrose, a cheap glucosyl donor substrate. The GTF-D mutant M4 catalyzed the glucosylation of a series of flavonoid compounds including genistein, daidzein, catechin and silybin. We observed a glucosylation bias of C-7 and C-4′ hydroxyl groups. C-5 and C-3′ hydroxyl-glucosylation products were not obtained. The major products were monoglucosylated, and only minor amounts of diglucosylated products were formed. In particular, all glucosylation products were α-configurated, thus it is now possible to study the bioactivities of various α-glucosylated flavonoids that do not exist in nature. The position of conjugation of the sugar moiety has a significant impact on the biological activity of natural products as well as their potential human health benefits^44^,^49^. The properties of natural products are also influenced by the regioselectivity of glycosyl conjugation. Some α-type natural product glycosides display unique properties in comparison to their β-anomers such as better inhibitory effects, less bitterness as sweeter, or higher solubility^50^,^51^,^52^. Our mutant enzyme can synthesize various α-glucosylated flavonoids, suggesting further novel properties of α-glucosylated natural products may be discovered in the future.

Glycosylation of the flavonoid catechin is also mediated by glucansucrases using sucrose or starch as the glucosyl donor^34^. However, multiple glucosylation products were obtained, including monoglucosyl and oligoglucosyl products. It has also been previously reported that wild-type GTF-D catalyzed the transglycosylation of catechin, resulting in catechin-4′-*O-*α-d-glucopyranoside (C1) and catechin-4′,7-*O-*α-di-d-glucopyranoside (C2)^40^. We also obtained these two products from the reaction catalyzed by the wild-type enzyme on catechin (Fig. 4D). However, the glucosylation product of catechin formed by mutant M4 was mainly catechin-4′-*O-*α-d-glucopyranoside which exceeded 90% of the total glucosylation products, greatly facilitating the downstream product purification process.

Nevertheless, the glucosylation reactions catalyzed by glucansucrases still suffer from the low thermodynamic favorability. The apparent equilibrium constant for the 4-MU glucosylation reaction in our study was estimated to be ~0.015^53^. Generally, an overdose of sugar donor sucrose needs to be supplemented to drive the reaction. Furthermore, to increase the production of phenolic glycoside products, attempts, such as removing fructose product and increasing the concentration of phenol substrates by optimizing reaction conditions, have been reported^39^,^40^. However, as a cheap, stable and easily-obtained donor, sucrose is still advantageous under some circumstances, compared with NDP-glucose.

In conclusion, by engineering the substrate promiscuity of glucansucrase GTF-D, the enzyme gained significantly improved capability to transfer the glucosyl moiety to a serious of non-glycosylated flavonoids by using sucrose, a cheap donor substrate. We thus demonstrated the feasibility of developing natural product glycosyltransferases by evolving transglycosidases using donor substrates other than NDP-sugars. The GTF-D mutant enzyme developed in this study has potential applications in glycodiversification studies.

## Methods

### General

Restriction enzymes, DNA polymerases and T4 DNA ligase were purchased from New England Biolabs (Beijing, China). Oligonucleotides was synthesized by Life Technologies (Shanghai, China). 4-MU and 4-MUG were purchased from Sigma-Aldrich (St. Louis, USA). (+)-Catechin, daidzein, genistein, genistin and silybin were purchased from Aladdin (Shanghai, China).

All *E. coli* strains were routinely grown in Luria-Bertani (LB) medium at 37 ^o^C. The antibiotics ampicillin (100 μg/mL) and kanamycin (50 μg/mL) were supplemented when necessary. The genomic DNA of *S. mutans* UA159 was kindly provided by Prof. Xiuzhu Dong from Institute of Microbiology, Chinese Academy of Sciences.

### Plasmid construction

A constitutive promoter P_BLMA_^54^ was inserted into vector pRX2 (http://www.addgene.org/vector-database/4032/) between the restriction sites *Xho*I and *Nco*I, which was designated as pRBH vector. The DNA sequence encoding the truncated GTF-D (GenBank accession number: AJD55265) without the predicted signal peptide (N-terminal 150 amino acids were truncated) was amplified by primers GTF-*Nco*I-fwd and GTF-*EcoR*I-rev using the genomic DNA of *S. mutans* UA159 as template. The PCR product was then subcloned downstream of P_BLMA_ promoter after digestion with *Nco*I and *EcoR*I, resulting in plasmid pRBH-GTF-D. For high-level expression and protein purification, the DNA sequence encoding truncated GTF-D was amplified with primers GTF-D-pET-*BamH*I-fwd and GTF-D-pET-*EcoR*I-rev and inserted to pET28a (Novagen) after digestion with *BamH*I and *EcoR*I, resulting in plasmid pET-GTF-D. See Table S1 for primer sequences used in this study.

### Construction of site-saturation mutagenesis library

Site-saturation mutagenesis library was constructed as described previously^55^. PCR was performed using pRBH-GTF-D as template with primers GTF-418-fwd and GTF-469-rev. Then the PCR product was used as mega-primer to perform megaprimer PCR of whole plasmids (MEGAWHOP) method using pRBH-GTF-D as template as described^56^. Following the MEGAWHOP PCR, *Dpn*I digestion (20 U) of the template was performed at 37 °C for 12 h, then *Dpn*I was inactivated at 80 ^o^C for 20 min. The PCR products were transformed into *E. coli* MC1061 and around 1,000 transformants were recovered. Ten randomly picked clones were sequenced, and these sequences revealed the expected random mutations at the targeted nucleotide positions, with no additional point mutations. Site-directed mutagenesis was performed using a QuikChange kit (Stratagene, La Jolla, USA).

### Library screening

The site-saturation mutagenesis library was screened as described^4^,^5^, with some modifications. Single colonies harboring the library mutants were grown in 1 mL LB medium supplemented with ampicillin in 96-well plates at 37 °C for 14 h. Cells were harvested by centrifugation (1,278 ×g, 10 min, 4 °C), then resuspended with 0.3 mL lysis buffer (50 mM Tris-HCl, 10 mg/mL lysozyme, pH 8.0) and incubated at 37 °C for 60 min. The cell debris were removed by centrifugation (1,840 ×g, 10 min, 4 °C) and the crude enzyme extracts were used for downstream enzymatic reactions. Enzyme assays were carried out by incubating 50 μL of crude enzyme extracts with 50 μL of the substrate solution (100 mM potassium phosphate buffer, 0.2 mM 4-MU, 200 mM sucrose, pH 6.0) in 96-well plates at 37 ^o^C for 5 h. Fluorescence of each well (excitation at 350 nm and emission at 460 nm wavelength) was determined both before and after the incubation with a SynergyMx Multi-Mode Microplate Reader (BioTek, Vermont, USA). The fluorescence differences of the variants between 0 and 5 h were calculated and the mutants with higher fluorescence decrease than the wild-type enzyme were selected for rescreening. The selected mutants were re-cultured in LB and the crude enzyme extracts were used to react with 4-MU and sucrose, and the productions of 4-MUG were determined with HPLC as described below.

### Protein purification

A single colony of strain BL21(DE3) harboring plasmid carrying gene of wild-type or mutant GTF-D was grown in LB medium at 37 °C and induced with 0.4 mM IPTG when OD_600_ reached 0.6, then the culture was continuously grown at 30 °C for 16 h. Cells were harvested by centrifugation at 4 °C, 3000 ×g for 20 min. The cells were then resuspended in the lysis buffer (50 mM Tris-HCl, 300 mM NaCl, 10 mM imidazole, pH 8.0) and disrupted by sonication with a JY92-IIN Ultra Sonic Cell Crusher (Ningbo, China). Cell debris were removed by centrifugation (15,000 ×g, 20 min, 4 °C) and the supernatants were loaded on a pre-equilibrated nickel-nitrilotriacetic acid (Ni-NTA) column (Qiagen, Valencia, USA). The column was washed with the lysis buffer and the bound protein was then eluted with the elution buffer (50 mM Tris-HCl, 300 mM NaCl, 200 mM imidazole, pH 8.0). Imidazole was removed by dialysis at 4 °C against 100 mM potassium phosphate buffer (pH 6.0). The purity of proteins were assessed by sodium dodecyl sulfate polyacrylamide gel electrophoresis (SDS-PAGE) and the protein concentrations were determined with Bradford method^57^.

### Enzyme assays

A standard enzyme reaction mixture included 150 μL purified enzyme (0.02 mg) mixed with 150 μL of substrate solution (20 mM for genistein or catechin and 10 mM for daidzein or silybin) in 100 mM potassium phosphate buffer (pH 6.0) containing 100 mM sucrose. The reaction mixtures were incubated at 37 °C for 5 h unless otherwise indicated and terminated by adding 300 μL of ice-cold methanol.

GTF-D transglycosylation products were determined with HPLC using a Shimadzu LC-20A system equipped with a photodiode array detector (Shimadzu Corp., Kyoto, Japan). LC-MS analysis was carried out by using an Agilent 1200 HPLC system and an Agilent Accurate-Mass-Q-TOF MS 6520 system equipped with an electrospray ionization source (Agilent Technologies, Santa Clara, USA). All MS experiments were detected in the positive ionization mode. A Waters Symmetry C18 column (250 × 4.6 mm, 5 μm) working at 45 °C was used for all analysis. For the products from 4-MU, genistein and daidzein, the mobile phase was 40–100% methanol (containing 0.1% formic acid) (0–15 min) at a flow rate of 0.8 mL/min, and the products were monitored at 260 nm. For the products from catechin and silybin, the mobile phase was 30% methanol (containing 0.1% formic acid) (0–15 min for catechin and 0–30 min for silybin) at a flow rate of 0.4 mL/min, and the products were monitored at 280 nm. The sugars were determined with HPLC using an Aminex HPX-87H Ion Exclusion Column (300 ×7.8 mm, Bio-Rad, USA) equipped with a refractive index detector (mobile phase consisted of 6 mM H_2_SO_4_ solution at a flow rate of 0.8 mL/min and temperature of 50 °C).

The kinetic parameters of GTF-D wild type and mutant enzymes were determined with the acceptor substrates genistein (2 ~ 8 mM) and catechin (2 ~ 8 mM) in the presence of 100 mM sucrose. The reaction products were analyzed with HPLC method described above. All assays were performed in three replicates and the kinetic parameters in Table 2 were obtained using Lineweaver-Burk plots.

### NMR spectroscopic analysis of transglycosylated products

^1^H, ^13^C and 2D NMR spectra of the purified transglycosylated products were recorded on a Brucker Avance 400 MHz instrument at 25 °C, using TMS as an internal standard.

### Determination of solubility and anti-oxidant activity

Aqueous solubility of the transglycosylated products were determined with a modified method^58^. For extensively and homogeneously mixing, sample solutions were maintained agitated (stirring) at 250 rpm at 25 °C for 24 h in a shaker. After this, the tubes were placed in a constant temperature thermostatic bath at 25 °C for 2 h. Then the samples were centrifuged at 17,000 ×g for 5 min, and the solution was tested with HPLC method mentioned above. All reported data in Fig. 4 represent the mean of three independent data points. The error bars represent standard deviations.

The anti-oxidation activity was tested with both the modified ferric ion reducing ability of plasma (FRAP) method^59^ and ferric thiocyanate method^60^,.

In the FRAP method, 150 μl of freshly prepared FRAP reagent was warmed to 37 °C, 50 μL sample was then added and the mixture was incubated at 37 °C for 10 min. The absorbance at 593 nm was measured with a SynergyMx Multi-Mode Microplate Reader (BioTek, Vermont, USA), and the background absorbance due to buffer served as the blank in all measurements. The anti-oxidant activity presented as the concentration of ferric reduced to ferrous form with a Fe^2+^ standard curve prepared in parallel (Figure S5). The anti-oxidant activities of the chemicals were defined as the concentrations of Fe^2+^ ions required for the equal anti-oxidant capability.

In the ferric thiocyanate method, 360 μL linoleic acid emulsion (prepared by homogenising 15.5 L of linoleic acid, 17.5 mg of tween-20 as emulsifier, and 5 mL phosphate buffer (pH 7.0)), 100 μL of 20 mM FeCl_2_, 100 μL of 30% NH_4_SCN and 40 μL sample was used. The 500 nm absorbance formed during linoleic acid peroxidation was measured every 12 h until reaching a maximum. The inhibition rate of lipid peroxidation in linoleic acid emulsion was calculated as follows:


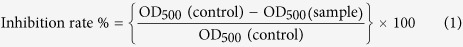


Buffer was used instead of sample in the control reaction (Table 3).

## Additional Information

**How to cite this article**: Liang, C. *et al.* Engineering a Carbohydrate-processing Transglycosidase into Glycosyltransferase for Natural Product Glycodiversification. *Sci. Rep.* **6**, 21051; doi: 10.1038/srep21051 (2016).

## Supplementary Material

Supplementary Information

## Figures and Tables

**Table 1:** Conversion rates of various acceptor substrates by wild-type and M4 mutant GTF-Ds.

Acceptors	Products	Conversion rate^a^ (nmol/min/mg)	
		Wild-type	M4 mutant
4-MU	4-MUG	9.8 ± 0.2	17.5 ± 0.1
Catechin	C1	7321.5 ± 2.2	12819.1 ± 130.6
Genistein	G1	0.3 ± 0.02	18.1 ± 0.1
	G2	0.3 ± 0.02	17.0 ± 0.2

^a^Rates of glucoside formation are shown in nanomoles of product formed per minute per mg of enzyme.

**Table 2:** Kinetic parameters of GTF-D wild-type and mutant M4 enzymes.

		*K*_m_ (mM)	*k*_cat_(min^−1^)	*k*_cat_/*K*_m_(min^−1^mM^−1^)
Catechin	WT	3.60 ± 0.44	546.35 ± 51.48	151.76
	M4	2.84 ± 0.37	738.15 ± 54.95	259.91
Genistein	WT	n.d.	n.d.	n.d.
	M4	1.47 ± 0.15	46.52 ± 2.41	31.65

n.d. not determined.

**Table 3:** Anti-oxidant activities of catechin and its α-glucopyranoside.

Methods	Catechin	Catechin-4′-*O*-α-d-glucopyranoside
FRAP method [Fe^2+^] (μM)*	59.0 ± 2.8	113.5 ± 1.5
Ferric thiocyanate method Inhibition rate (%)**	81.4 ± 0.7	84.3 ± 1.1

^*^The standard curve used for FRAP method was shown in Figure S5.

^**^For Ferric thiocyanate method, the inhibition rates of lipid peroxidation in linoleic acid emulsion by the compounds at 60 h were calculated as described in supplementary materials (Figure S6).
