# Supplementary material for: Engineering a Carbohydrate-processing Transglycosidase into Glycosyltransferase for Natural Product Glycodiversification
Source: Sci Rep. 2016 Feb 12;6:21051. doi: 10.1038/srep21051 (PMC4751530; doi:10.1038/srep21051)
Supplement: Supplementary Information [file srep21051-s1.pdf]

Supplementary Information for:

Engineering a Carbohydrate-processing Transglycosidase into Glycosyltransferase for Natural Product Glycodiversification

Chaoning Liang,[1*] Yi Zhang,[1,5*] Yan Jia,[2] Wenzhao Wang,[4] Youhai Li,[3] Shikun Lu,[1,5] Jian-Ming Jin [2,3#] & Shuang-Yan Tang[1#]

[1]CAS Key Laboratory of Microbial Physiological and Metabolic Engineering, Institute of Microbiology, Chinese Academy of Sciences, Beijing 100101, China; [2]Beijing Key Laboratory of Plant Resources Research and Development, Beijing Technology and Business University, Beijing 100048, China; [3]School of Chemistry and Biotechnology, Yunnan Minzu University, Kunming, China; [4]State Key Laboratory of Mycology, Institute of Microbiology, Chinese Academy of Sciences, Beijing, China; [5]University of Chinese Academy of Sciences, Beijing, China.

* These authors contributed equally to this work.

Methods

Purification of transglycosylated products. 4.8 mg purified mutant M4 enzyme was mixed with 200 mg genistein in 80 mL of 100 mM potassium phosphate buffer (pH 6.0) containing 50% DMSO and 200 mM sucrose. The reaction mixtures were incubated at 37 ℃ for 5 h. Then the reaction product was concentrated under reduced pressure. The concentrated reaction product was subjected to semi-preparative HPLC (LC3000 HPLC system, Beijing Chuang Xin Tong Heng Science & Technology Co., Ltd, Beijing, China) on YMC ODS-A column with 50% methanol (containing 0.1% formic acid) and to yield G1 (6 mg) and G2 (5 mg).

1 mg purified mutant M4 enzyme was mixed with 46.4 mg catechin in 8 mL of 100 mM potassium phosphate buffer (pH 6.0) containing 200 mM sucrose. The reaction mixtures were incubated at 37 ℃ for 5 h. The catechin reaction product was concentrated by vacuum evaporation, then concentrated reaction product was subjected to semi-preparative HPLC on YMC ODS-A column with 30% methanol (containing 0.1% formic acid) and to give C1 (50 mg).

Homology modeling and docking study. Ten three-dimensional models were constructed using the academic version of MODELLER[1,4] with the default parameters based on the structure of *L. reuteri* 180 glucansucrase GTF180 (PDB ID 3HZ3)[2].The best model was chosen for further refinement by MD simulation.

The system energy was minimized with a harmonic constraint of 100 kJ/mol/Å2 for 5000 steps, including 2500 steps of steepest descent, followed by 2500 steps of conjugated gradient. After that, the system was heated from 0 to 300 K. The production simulation lasted for 6 ns at constant temperature (300 K) and pressure with a 1 fs time step. In all of the theoretical simulations, a 10 Å nonbonded cutoff was used. APARM03 force field was used for the enzyme using the Amber9 software package[3].

4-MU was docked into this model. The automatic docking calculations were done by the program AutoDock 4[3] and the best docked structure was selected.

Reference

1. Eswar, N. *et al.* Comparative protein structure modeling using modeller. *Curr. Protoc. Bioinform.* 5.6.1-5.6.30 (2006).
2. Marti-Renom, M.A. *et al.* Comparative protein structure modeling of genes and genomes. *Annu. Rev. Biophys.*

Biomol. Struct. **29**, 291–325 (2000).

3. Vujičić-Žagar, A. *et al.* Crystal structure of a 117 kDa glucansucrase fragment provides insight into evolution and product specificity of GH70 enzymes. *Proc. Natl. Acad.Sci. U.S. A.* **107**, 21406-21411 (2010).
4. Pearlman, D.A. *et al.* AMBER, a package of computer programs for applying molecular mechanics, normal mode analysis, molecular dynamics and free energy calculations to simulate the structural and energetic properties of molecules. *Comput. Phys. Commun.* **91**, 1–41 (1995).
5. Morris, G. M. *et al.* Automated docking using a Lamarckian genetic algorithm and an empirical binding free energy function. *J. Comput. Chem.* **19**, 1639-1662 (1998).
6. Monchois, V., Willemot, R.-M. & Monsan, P. Glucansucrases: mechanism of action and structure–function relationships. *FEMS Microb. Rev.* **23**, 131-151 (1999).

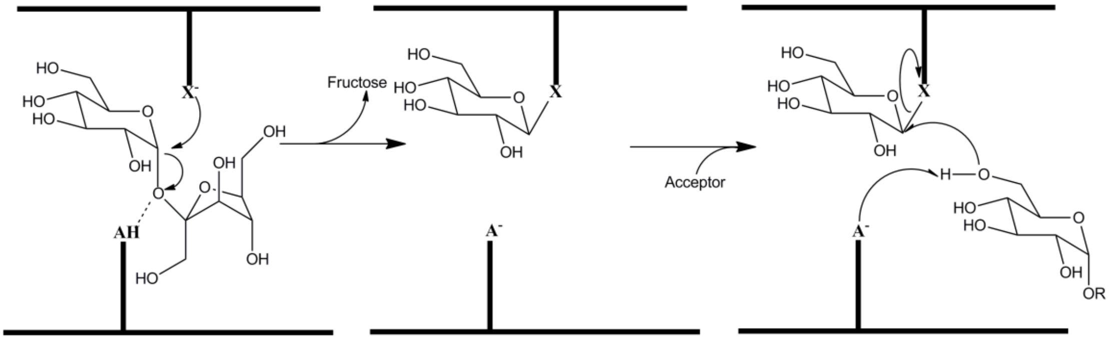

Figure S1. The catalytic mechanism of glucansucrase (adapted from reference 6).

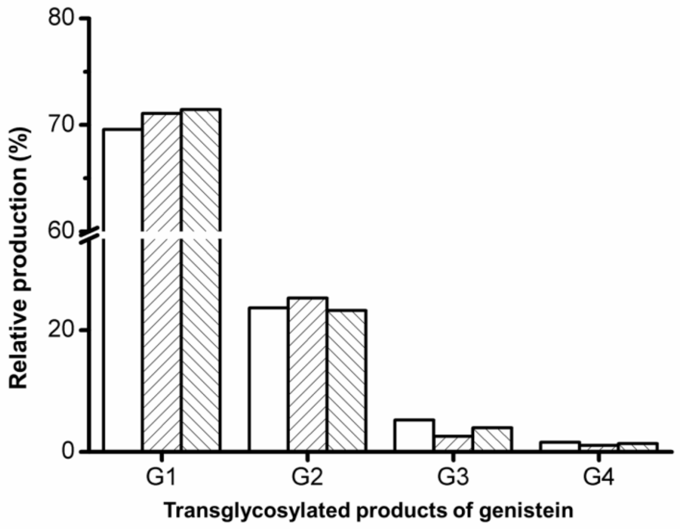

Figure S2. Relative productions of the transglycosylated products of genistein produced by GTF-D mutant M4 using various concentrations of sucrose (blank, 50 mM; left-slash, 100 mM; right-slash 200 mM) under standard reaction conditions. The relative production was calculated as the production percentage of individual transglycosylated product in the total transglycosylated products.

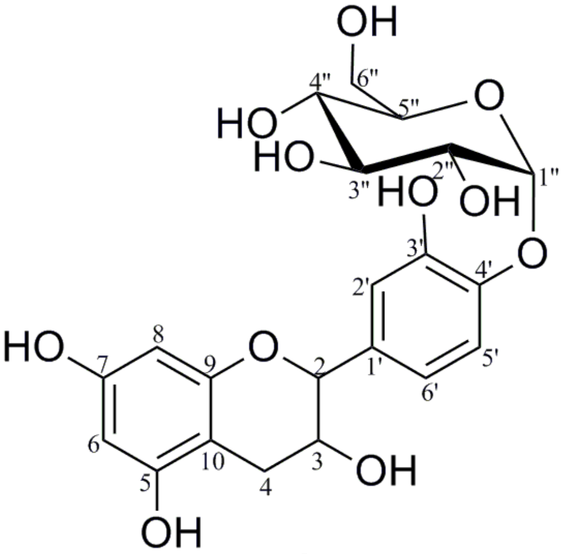

C1

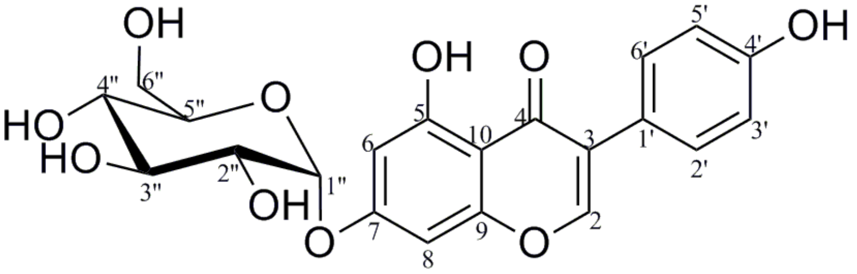

G1

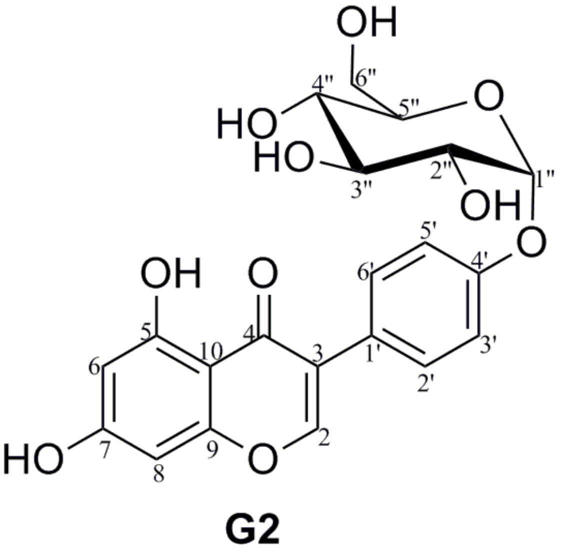

G2

Figure S3. Chemical structures of the glycoside products.

C1, catechin-4’-*O*-α-D-glucopyranoside; G1, genistein-7-*O*-α-D-glucopyranoside; G2, genistein-4'-*O*-α-D-glucopyranoside.

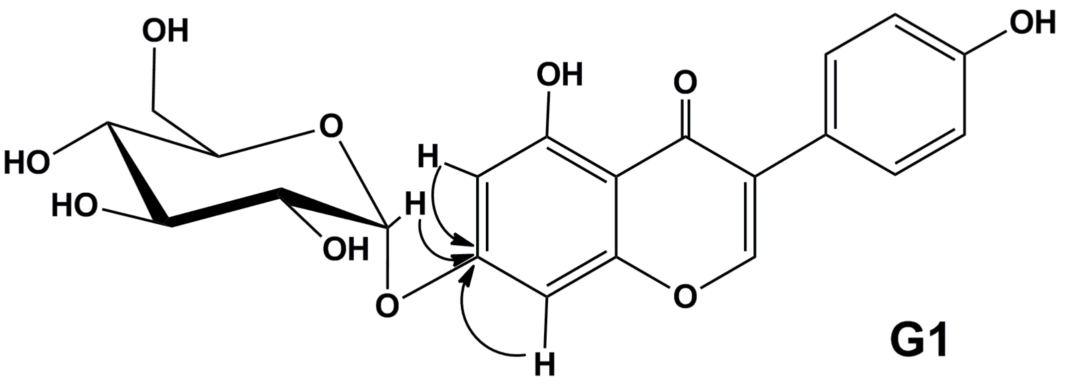

G1

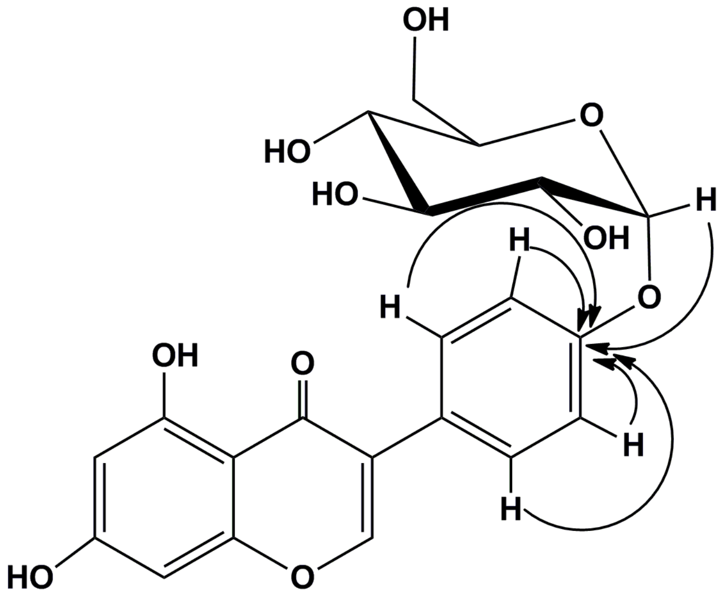

G2

Figure S4. Key HMBC correlations of G1 and G2.

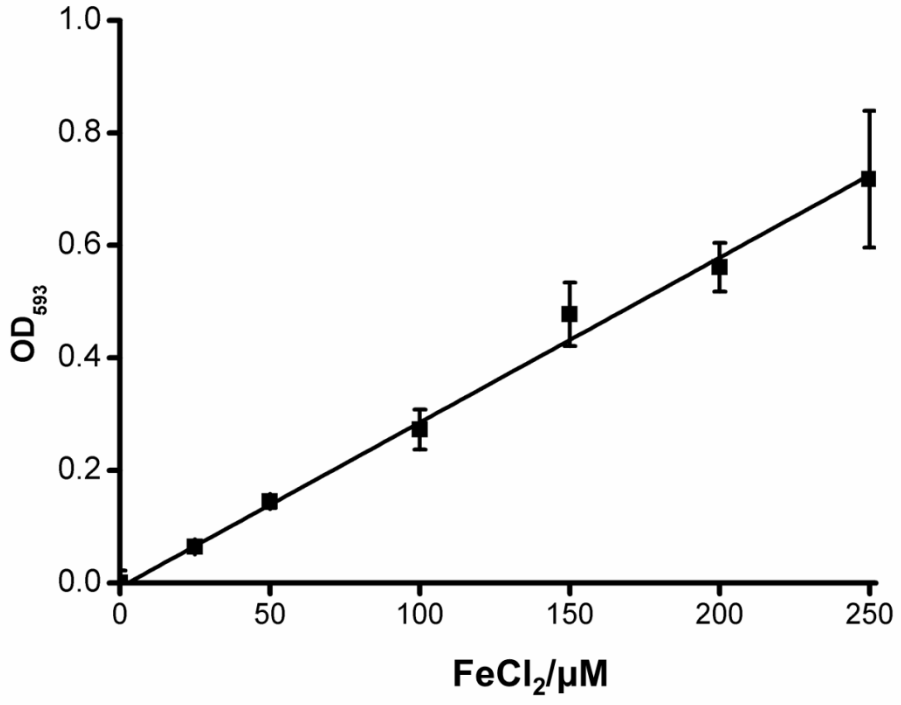

Figure S5. Standard curve for FRAP method, using $FeCl_2$ as standard solution as described in "Materials and Methods" section.

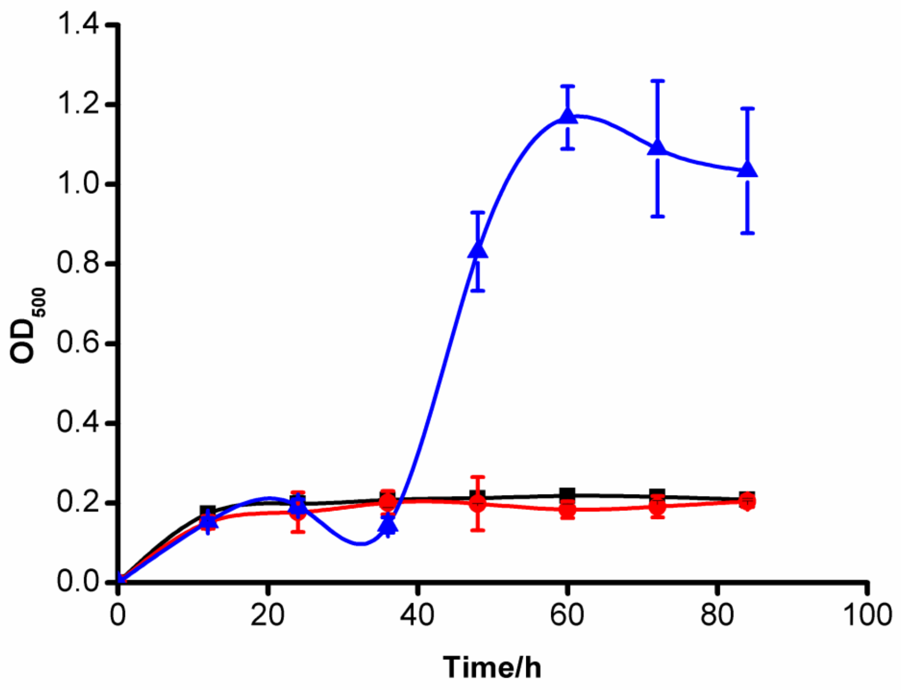

Figure S6. Anti-oxidant activities of catechin (black curve) or catechin-4’-*O*-α-D-glucopyranoside (red curve) at the concentration of 500 μM, tested with ferric thiocyanate method. Comparing with the control (blue curve), the inhibition rates of lipid peroxidation in linoleic acid emulsion by the compounds at 60 h were calculated as described in "Materials and Methods" section and presented in Table 3.

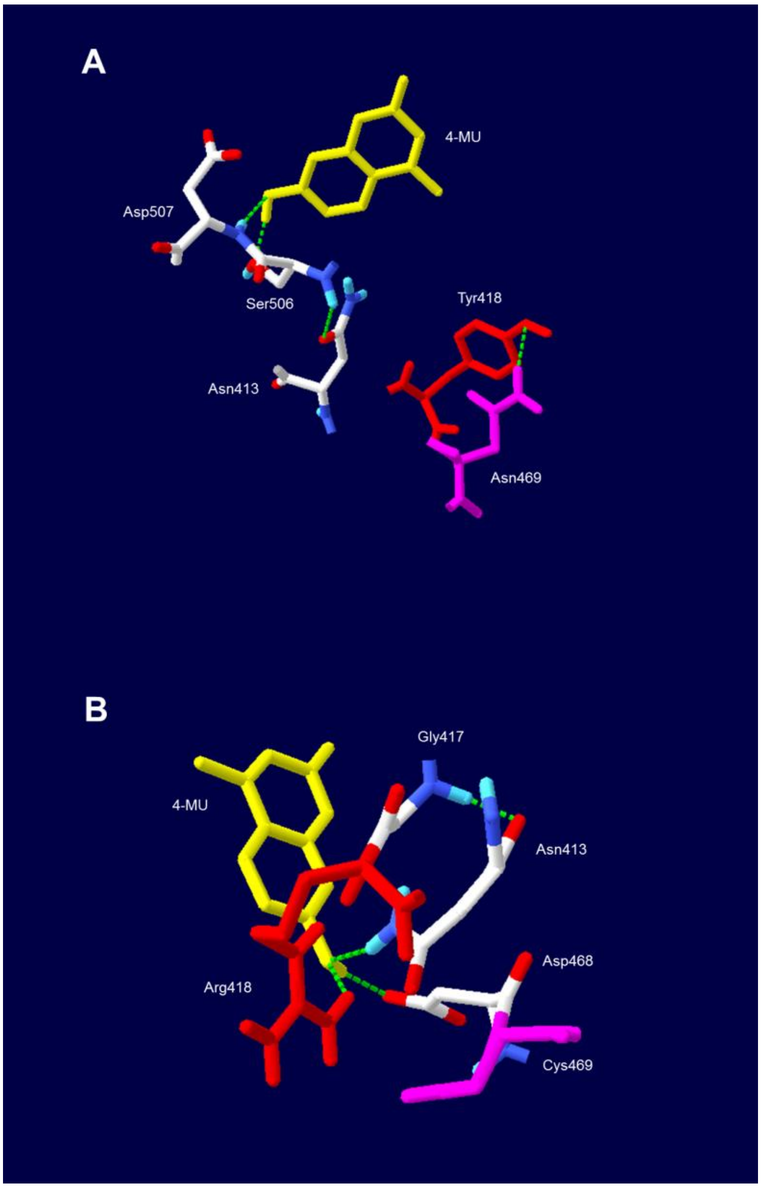

Figure S7. 4-MU docked into the acceptor binding pockets of the model structures of GTF-D wild type (A) and mutant M4 (B). 4-MU was colored in yellow, Try418 (Arg418 in M4) was displayed in red, Asn469 (Cys469 in M4) was colored in pink. The hydrogen bonding interactions was displayed in green.

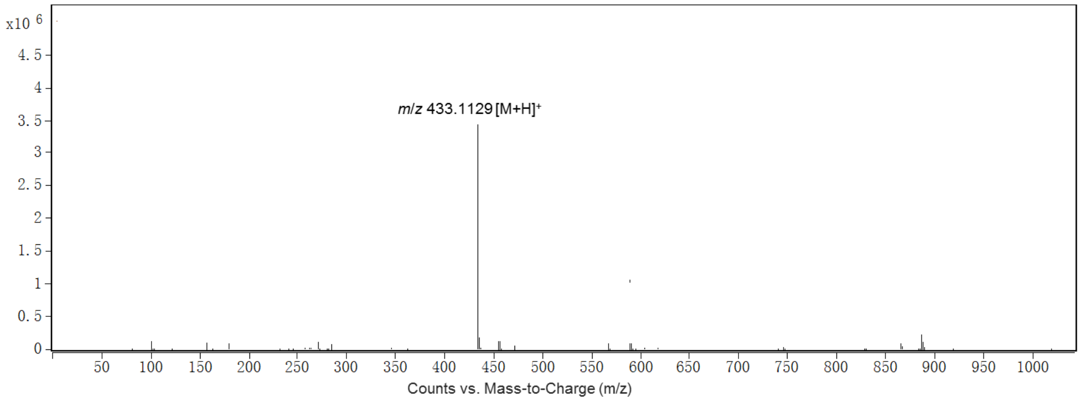

G1 HR-ESI-MS

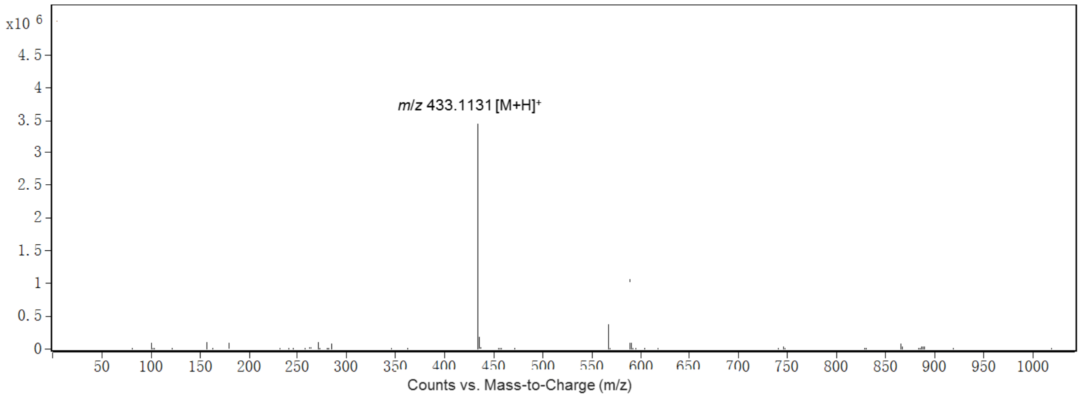

G2 HR-ESI-MS

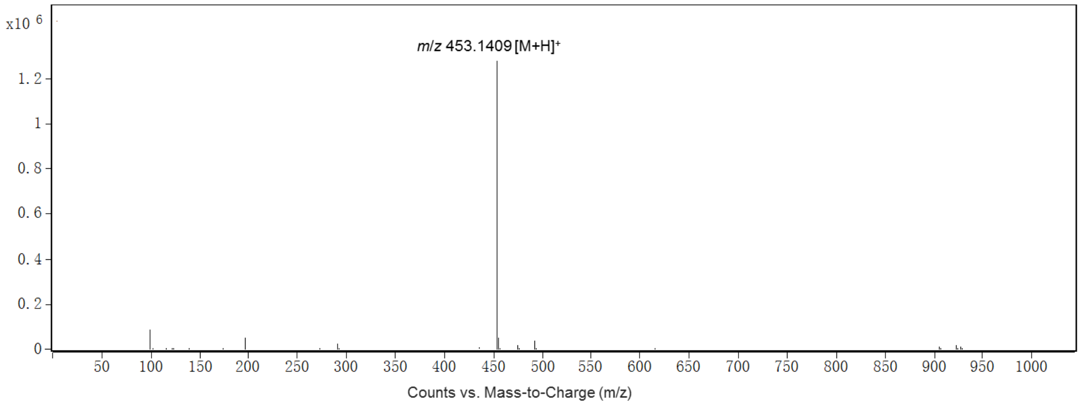

C1 HR-ESI-MS

Figure S8. HR-ESI-MS data of G1, G2 and C1.

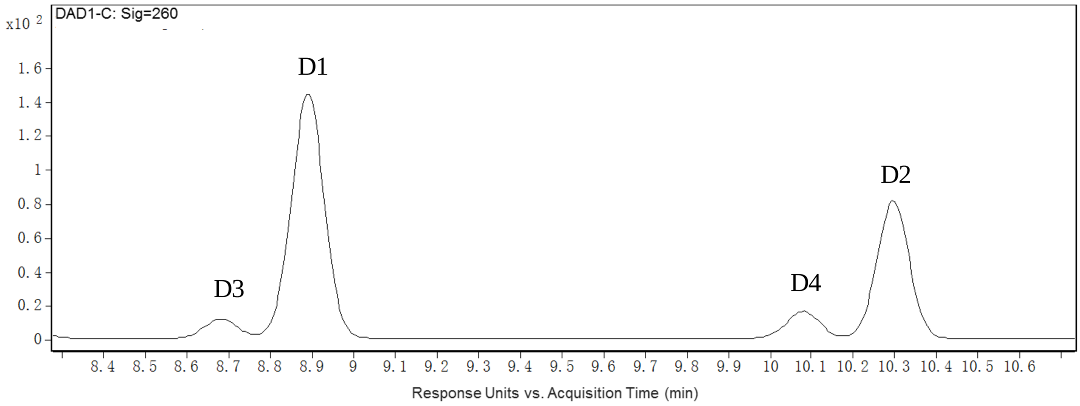

LC-UV of daidzein

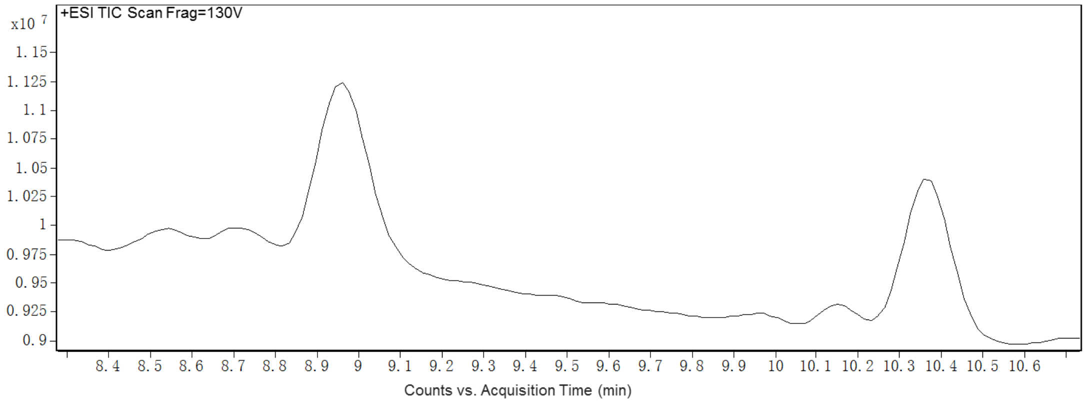

LC-MS of daidzein

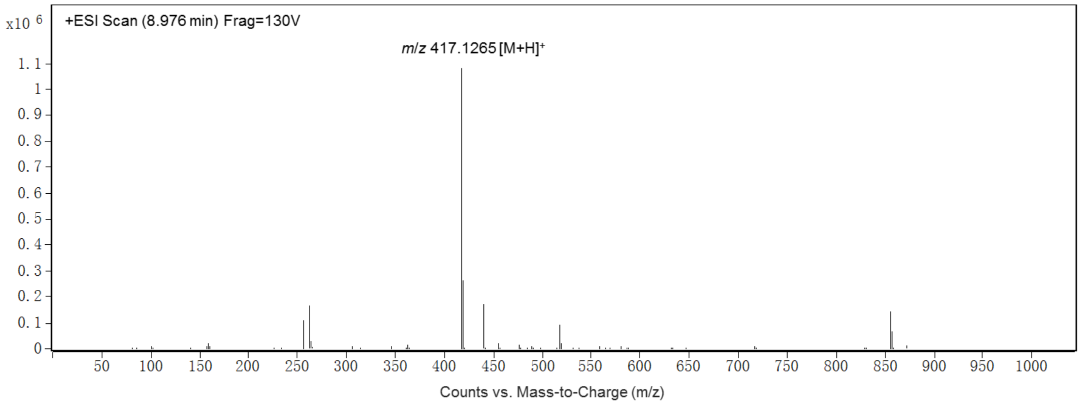

D1 MS

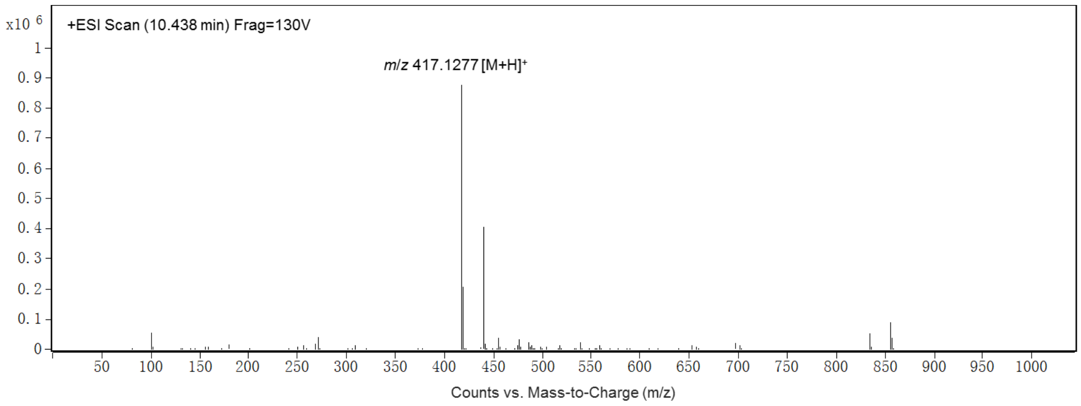

D2 MS

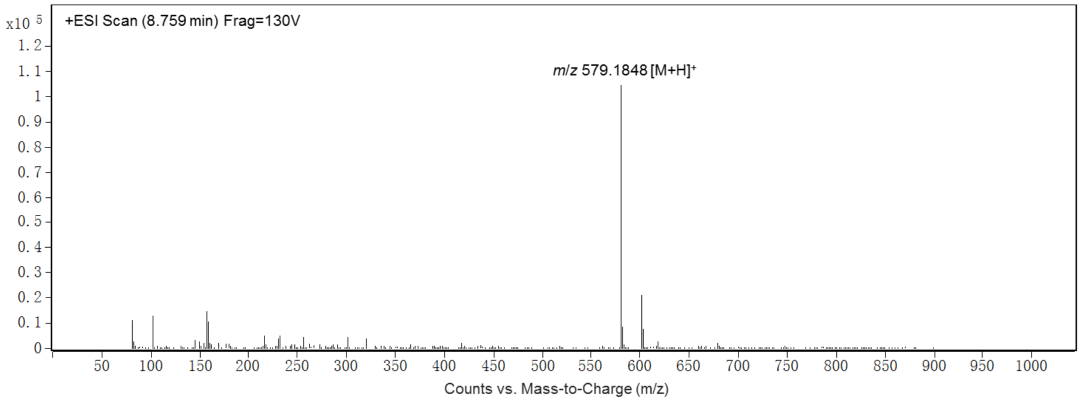

D3 MS

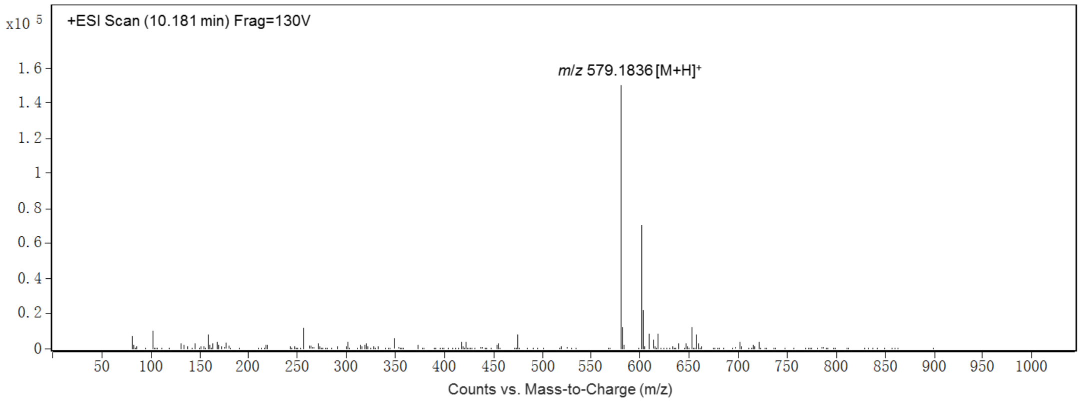

D4 MS

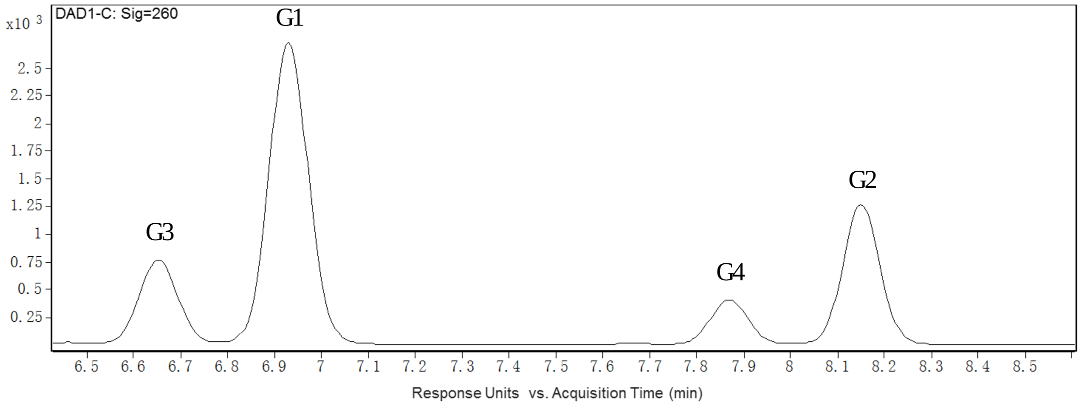

LC-UV of genistein

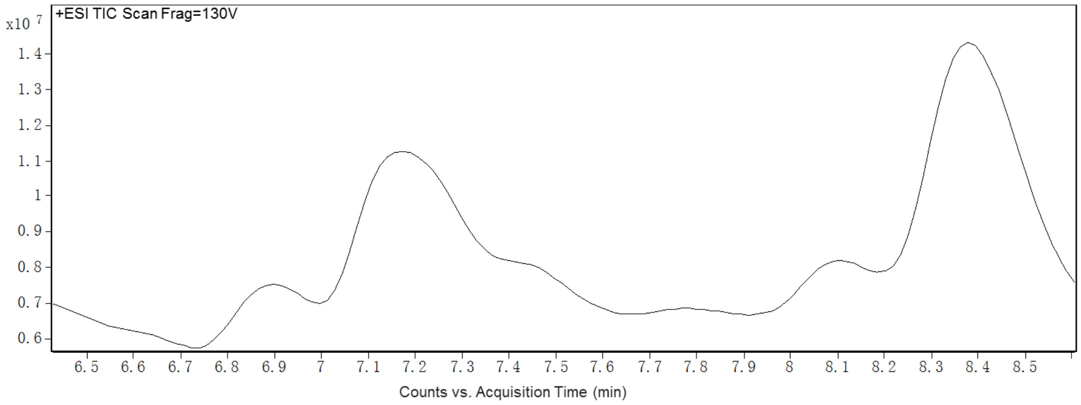

LC-MS of genistein

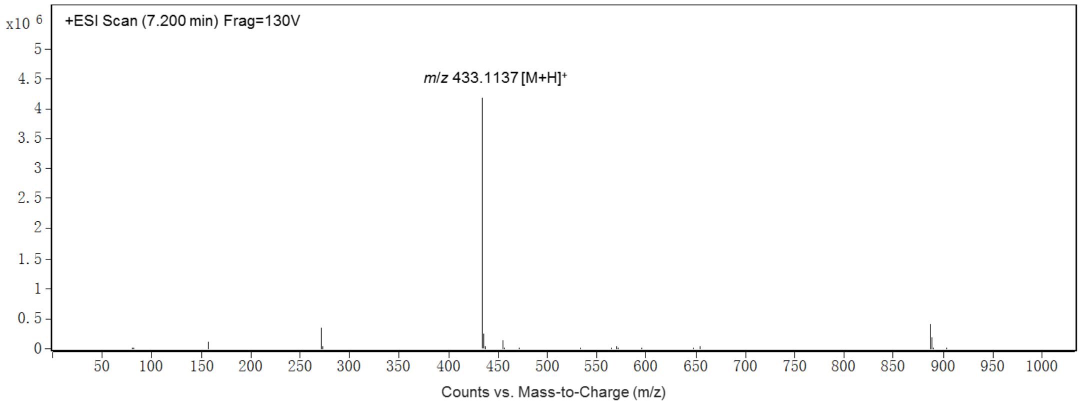

G1 MS

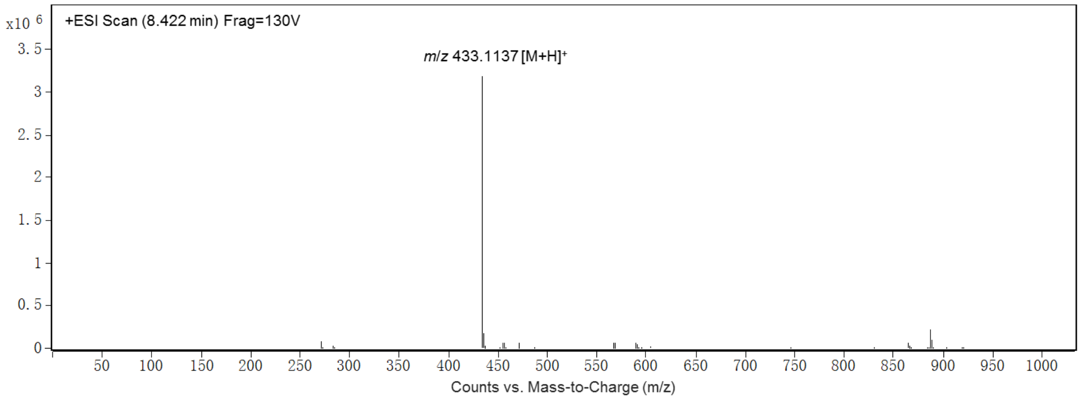

G2 MS

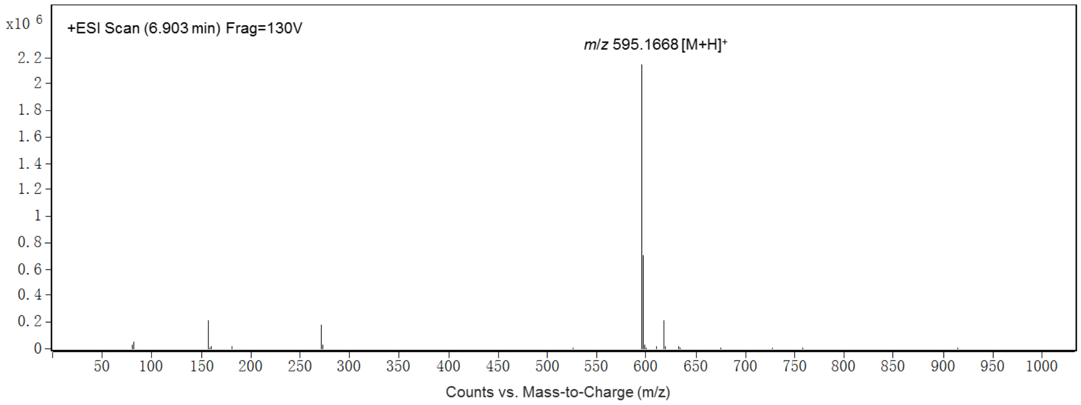

G3 MS

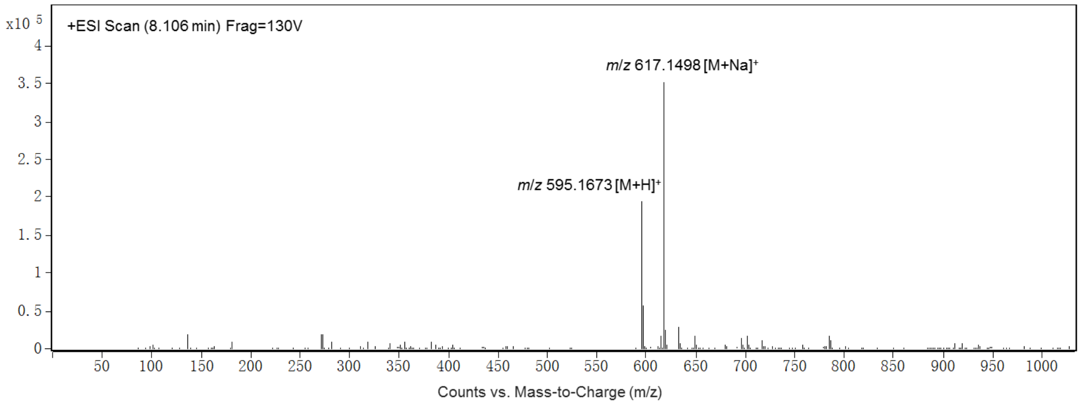

G4 MS

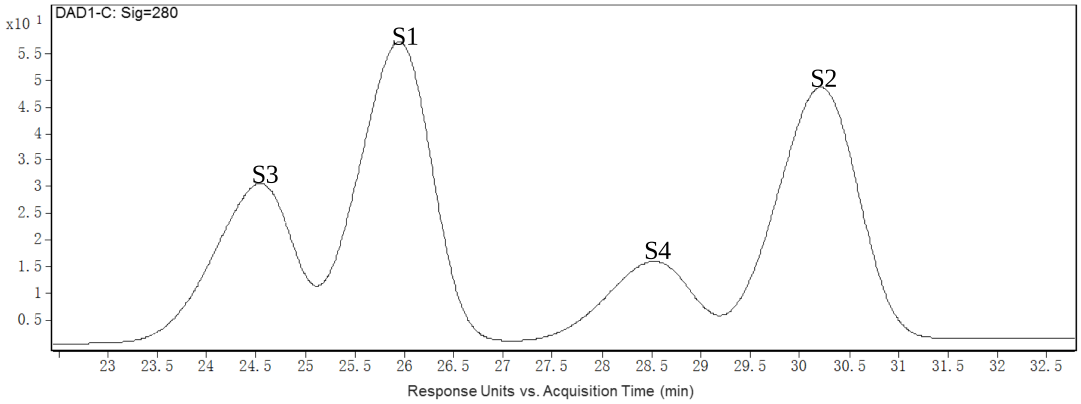

LC-UV of silybin

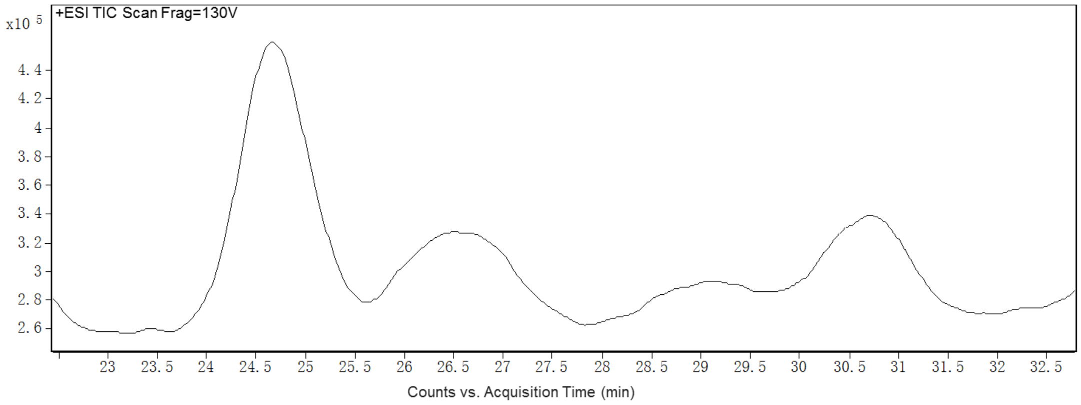

LC-MS of silybin

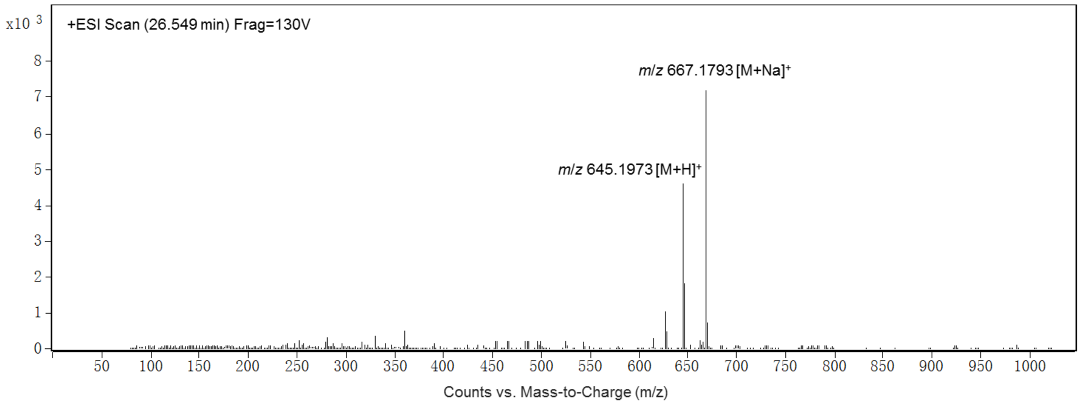

S1 MS

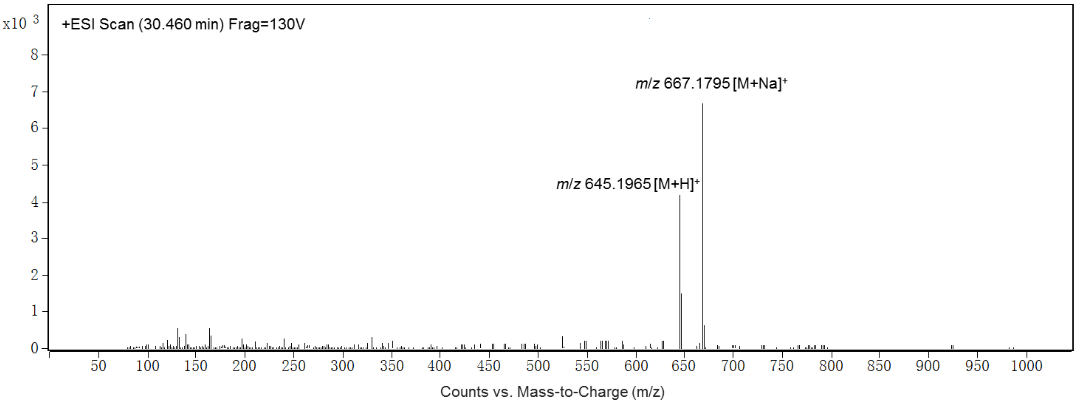

S2 MS

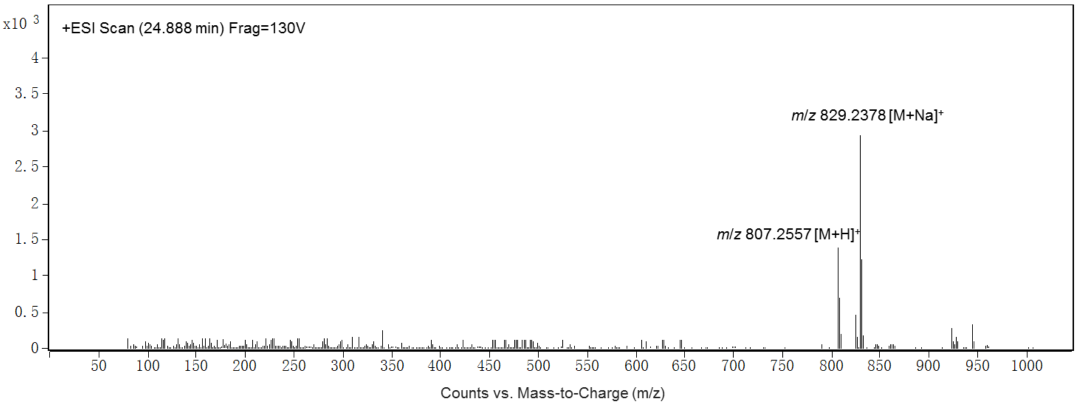

S3 MS

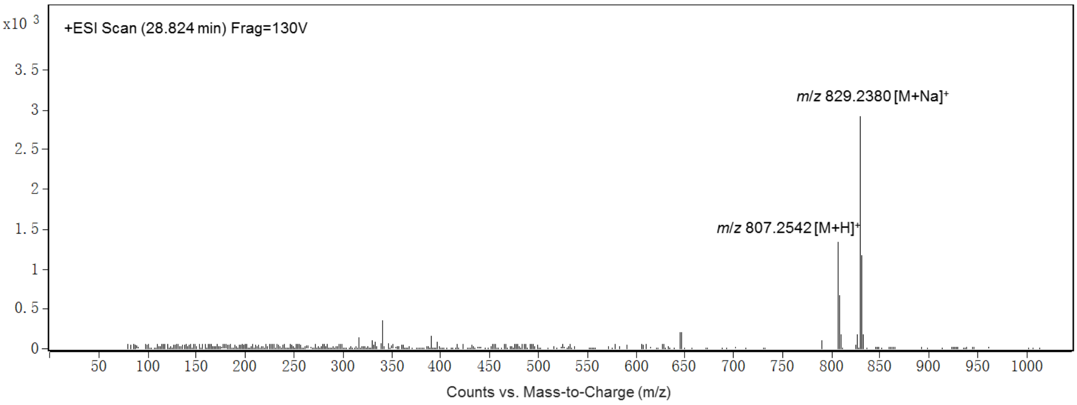

S4 MS

Figure S9. LC-MS data of the reaction mixtures of mutant M4 reacted with daidzein, geinistein, or silybin under standard reaction conditions.

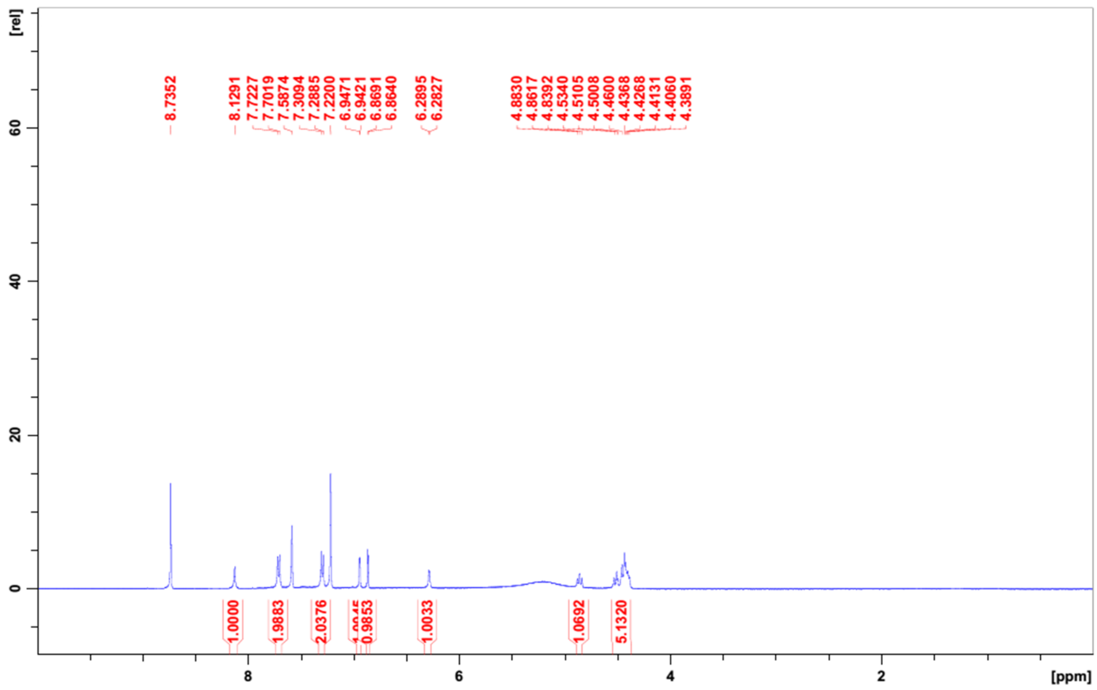

G1 ^{1}H spectrum

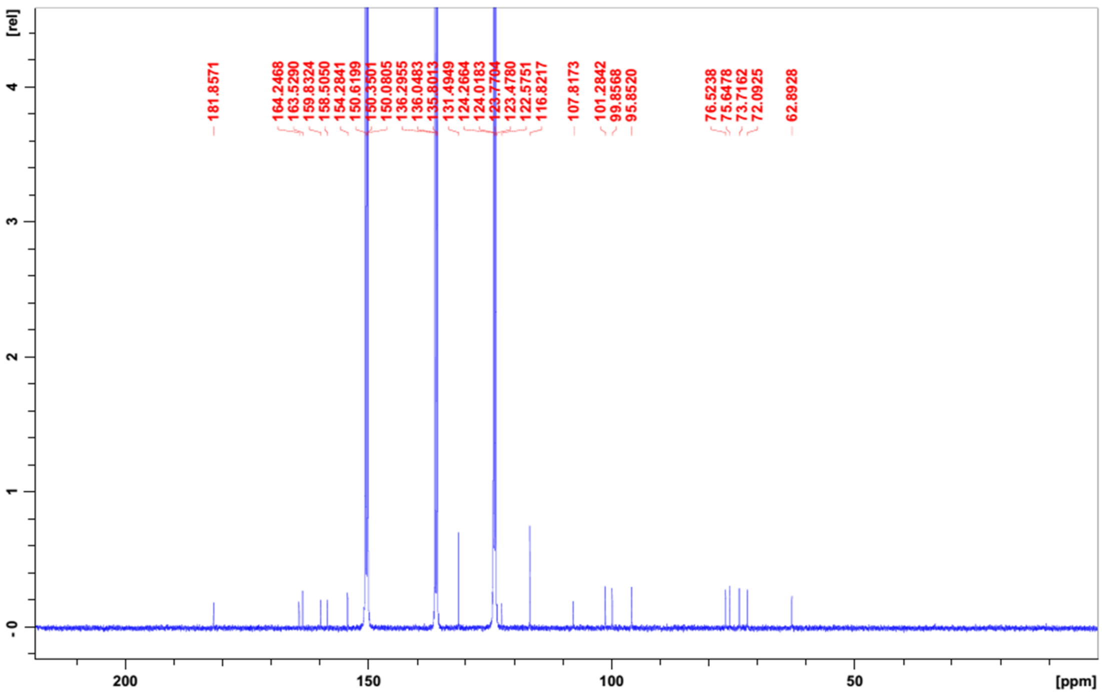

G1 ^{13}C spectrum

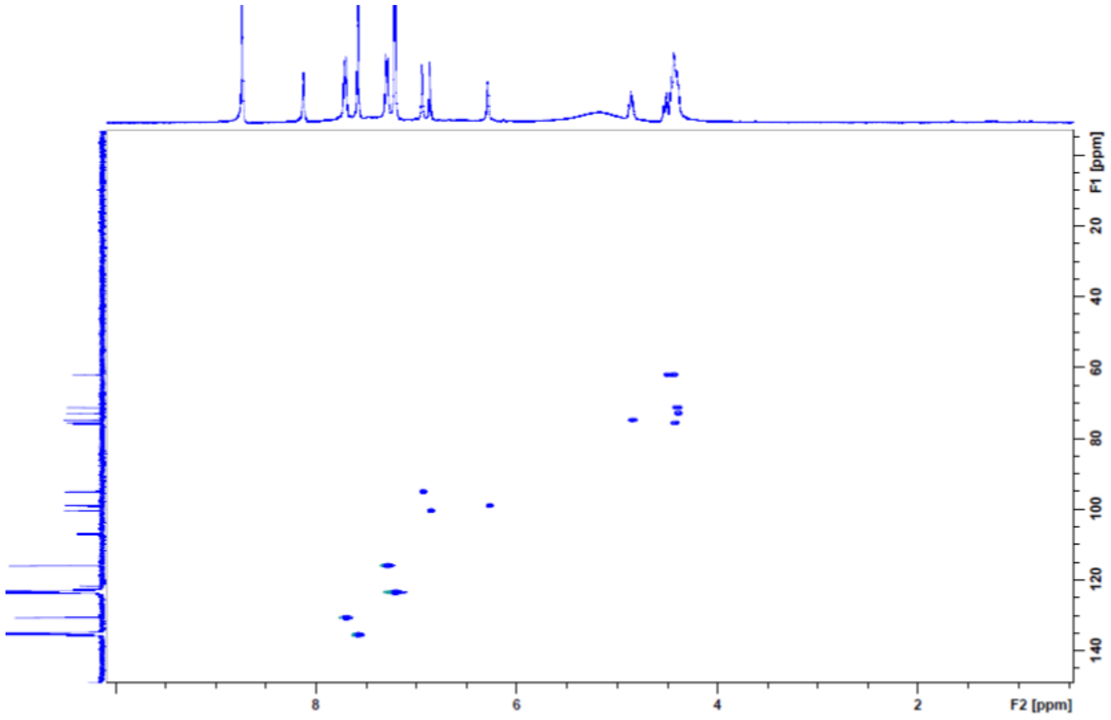

G1 HMQC spectrum

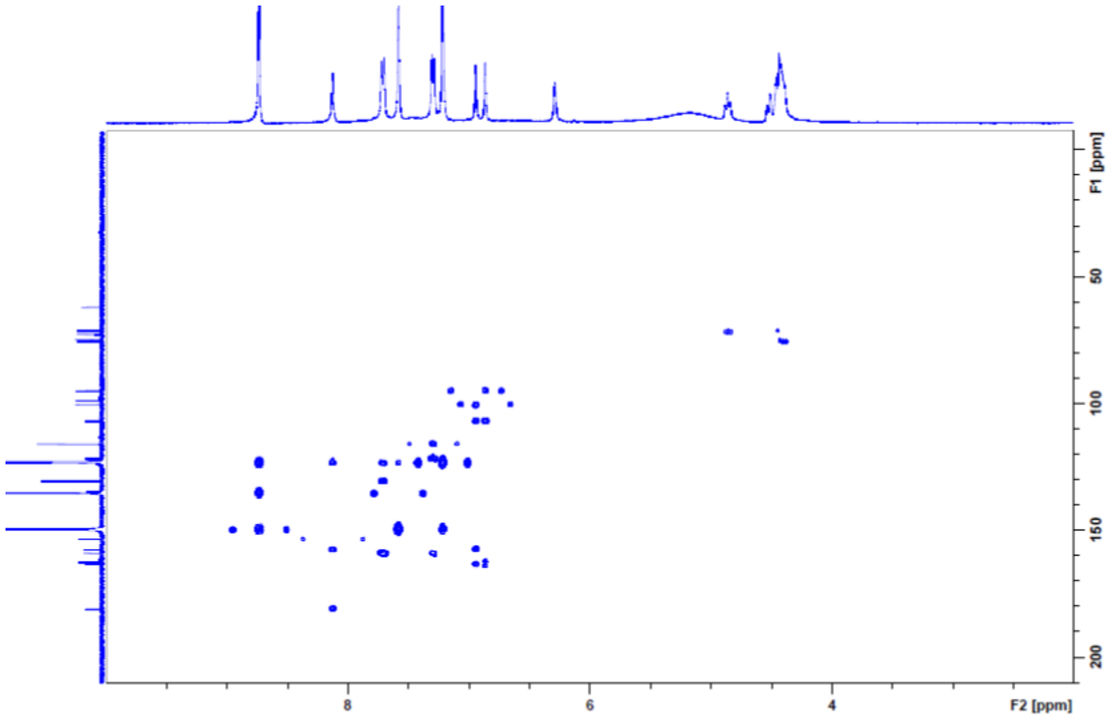

G1 HMBC spectrum

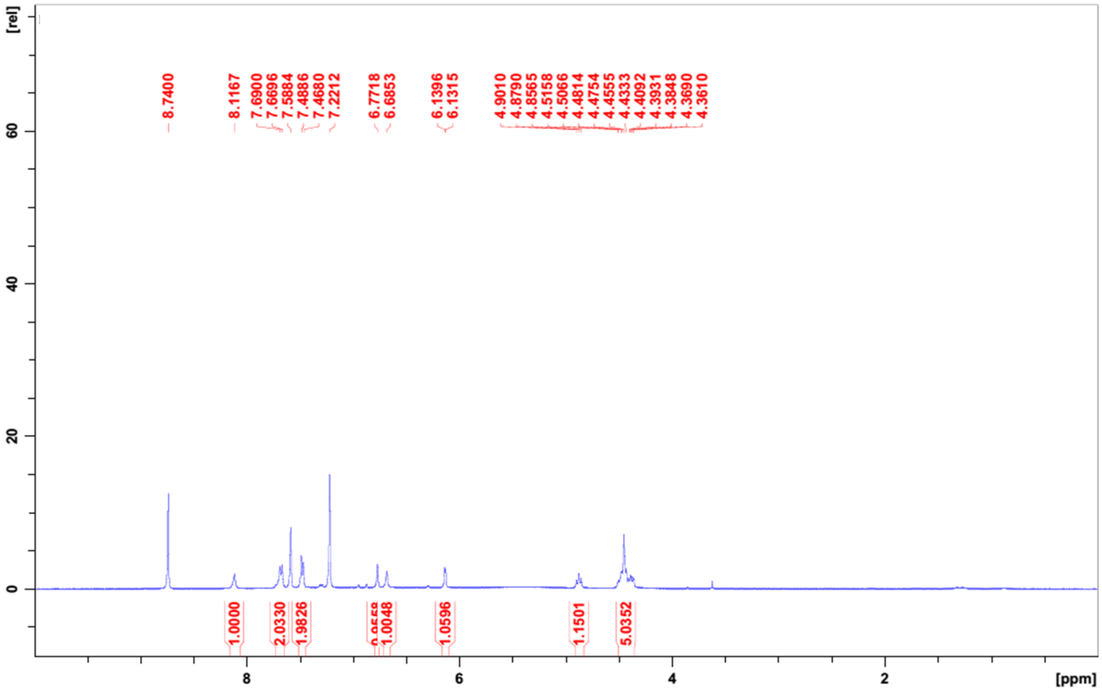

G2 ^{1}H spectrum

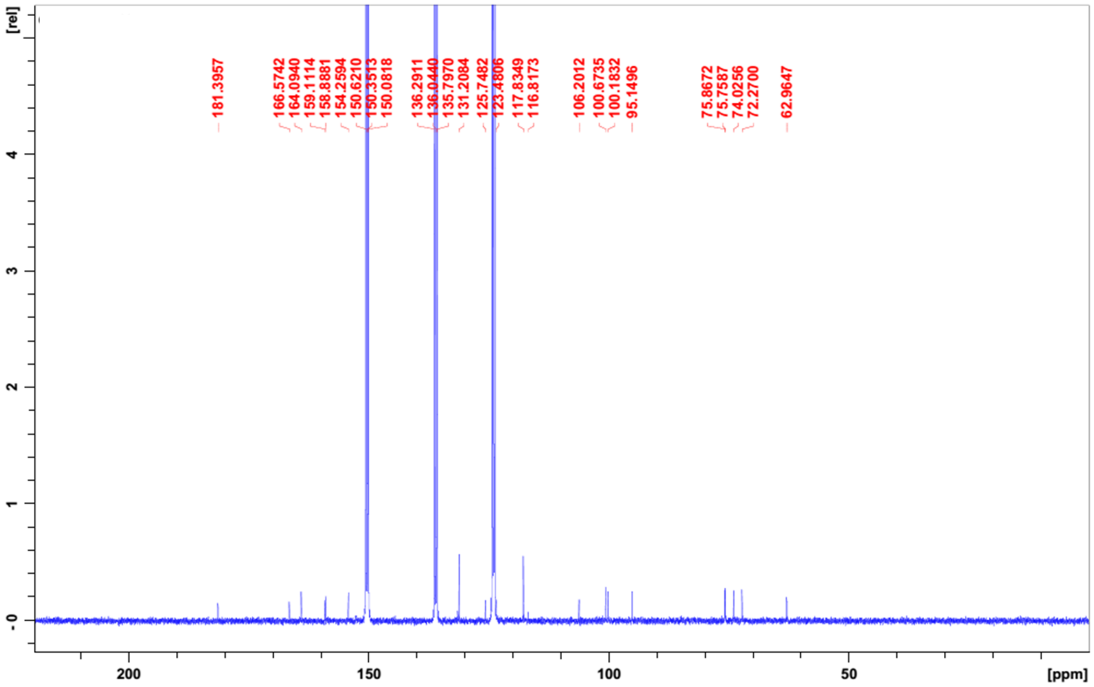

G2 ^{13}C spectrum

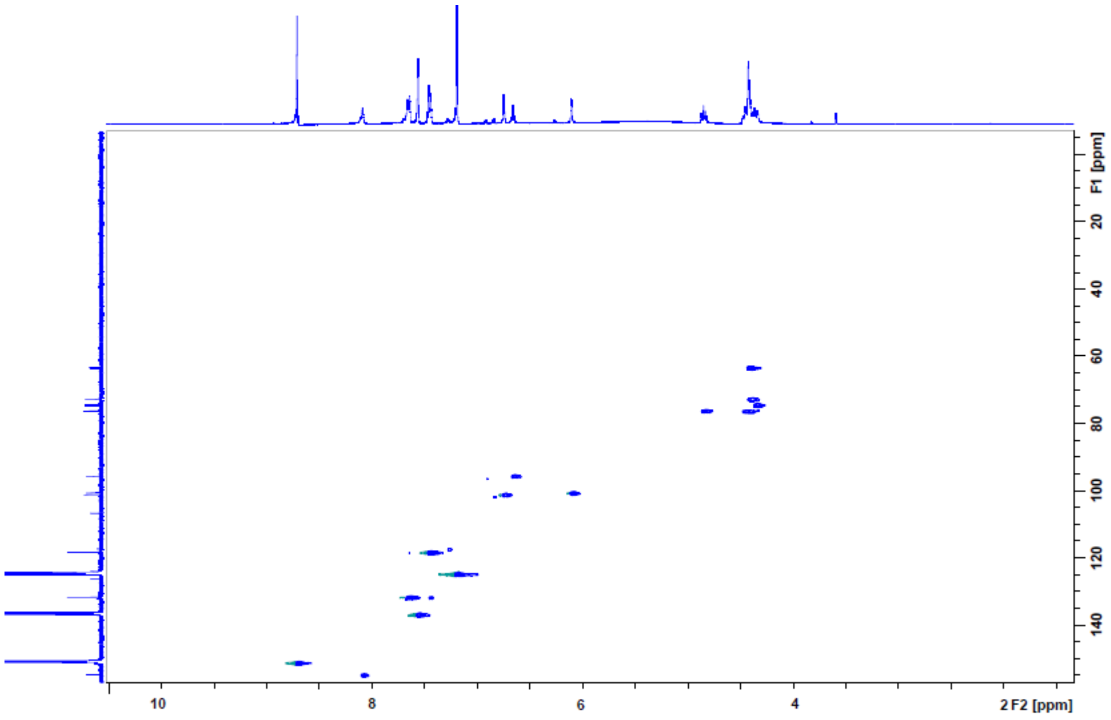

G2 HMQC spectrum

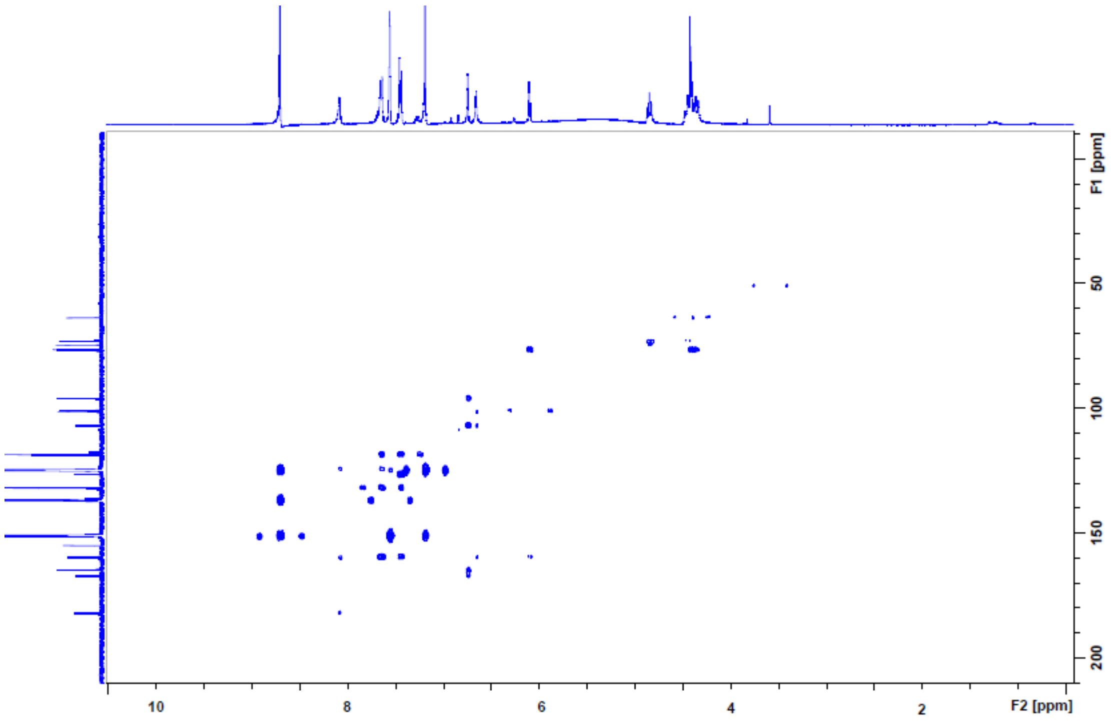

G2 HMBC spectrum

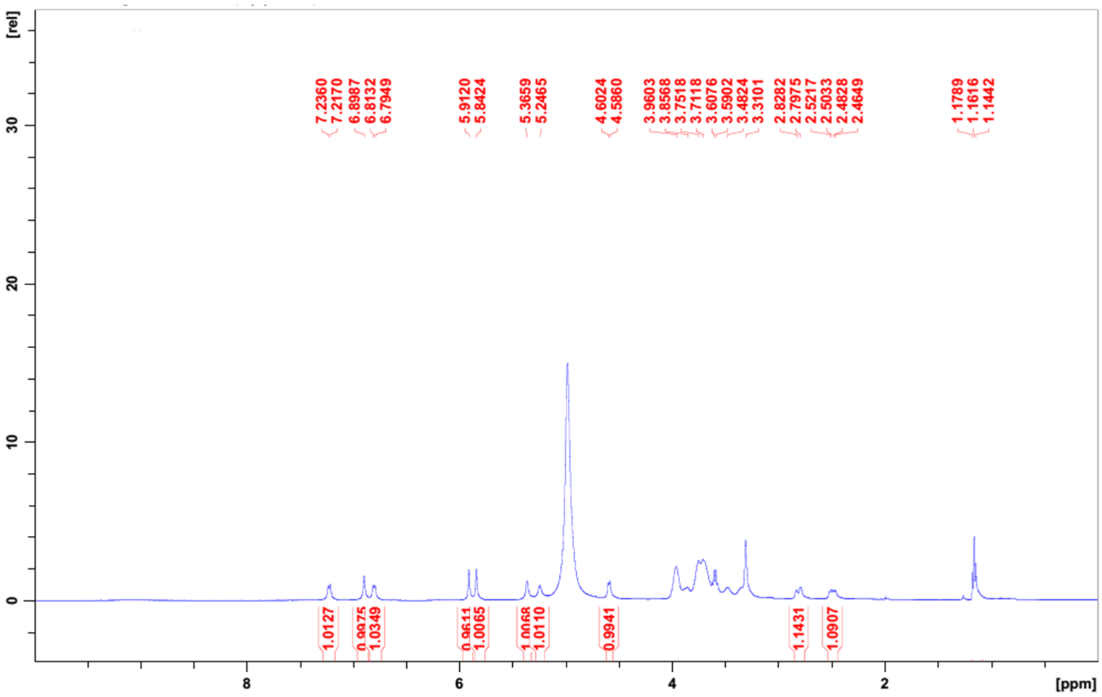

C1 ^{1}H spectrum

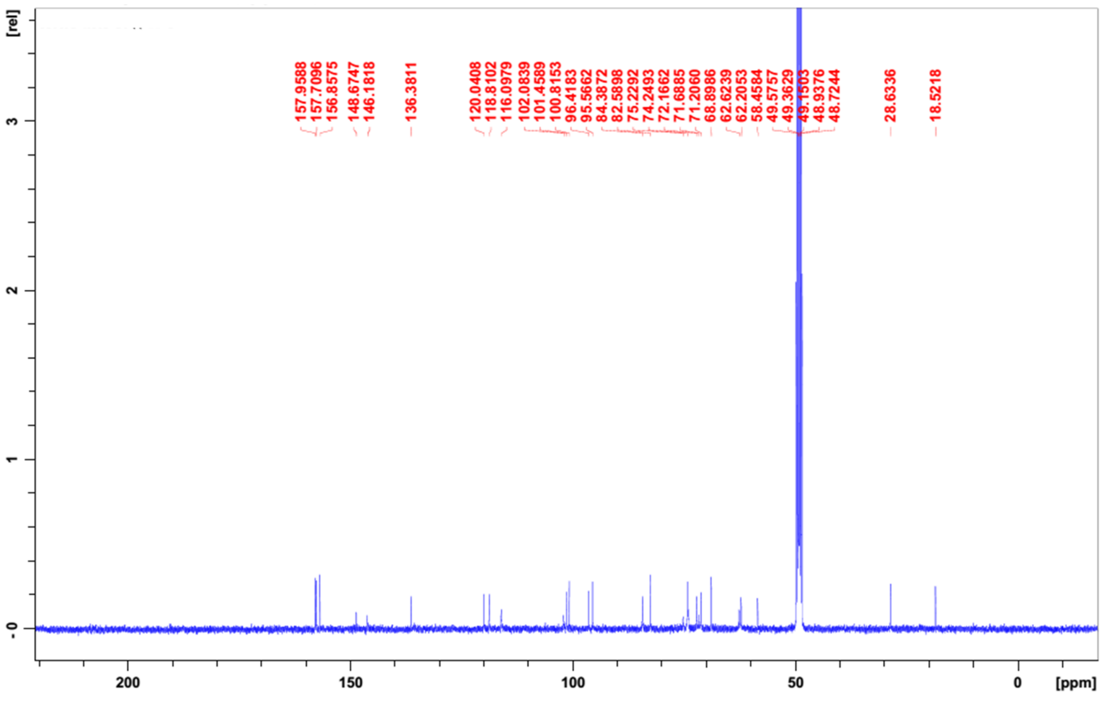

C1 ^{13}C spectrum

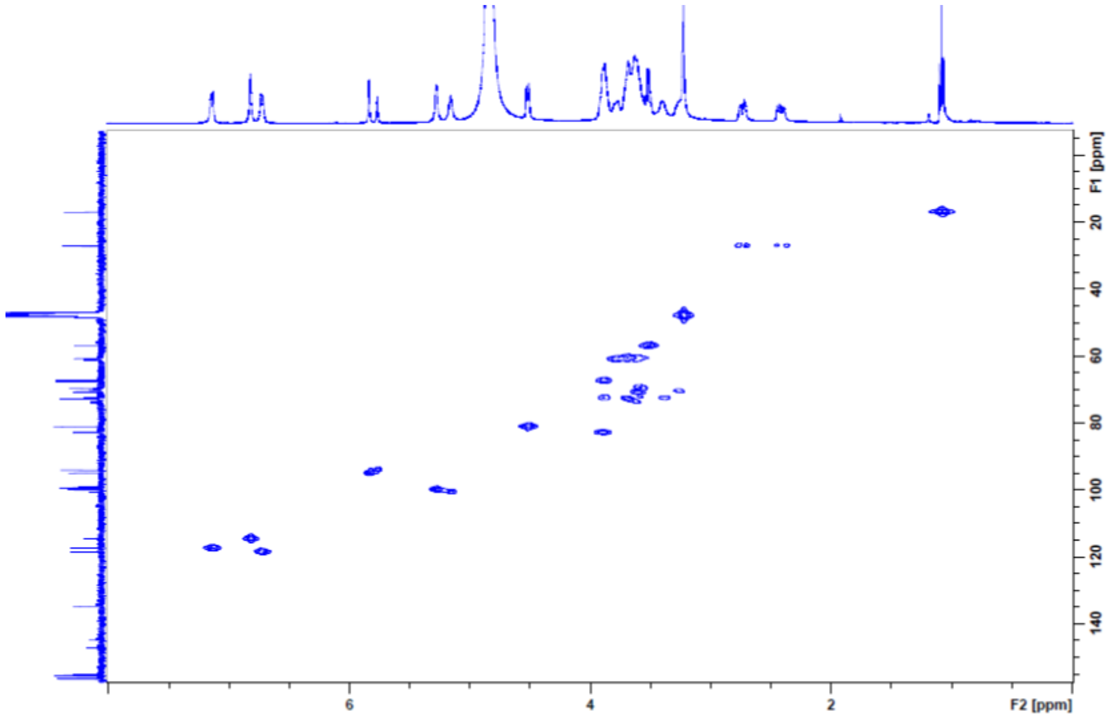

C1 HMQC spectrum

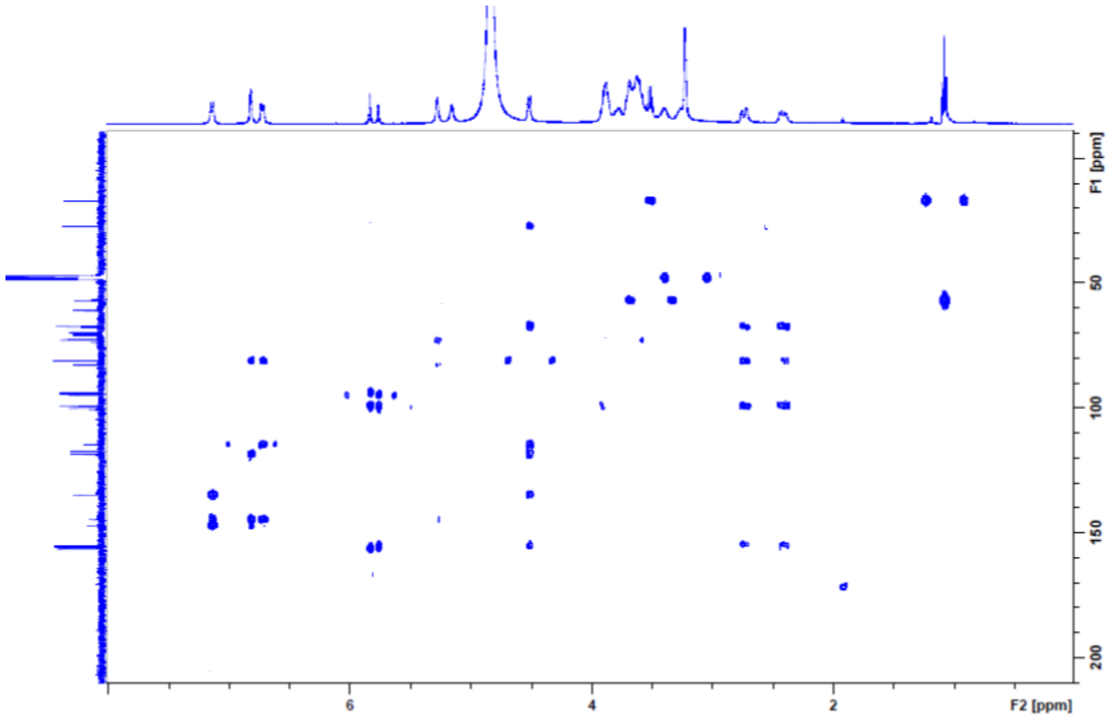

C1 HMBC spectrum

Figure S10. NMR spectra of G1, G2 and C1.

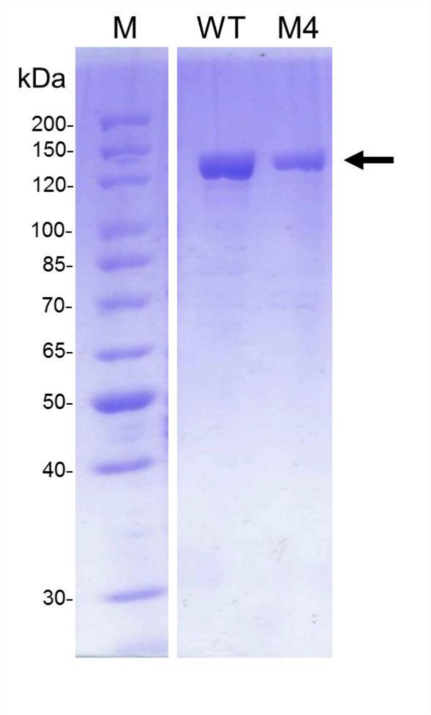

Figure S11. SDS-PAGE analysis of purified GTF-D enzymes wild type (WT) and M4 mutant.

Table S1 Primers used in this study

Primer	*Sequence (5'-3')*
pBLMA-*Xho*I-fwd	CCGCTCGAGAATTCGGAAGATGTCGCGGTC
pBLMA-*Nco*I-rev	GTTCCATGGTGTTTCCTCC
pRX-*Xho*I-fwd	CCGCTCGAGGGGATAACGCAGGAAAGAAC
pRX-*Nco*I-rev	CAGCCATGGATCCGACAGAACAGC
GTF-*Nco*I-fwd	TCTCCATGGACAGAACAGCAGACCTCAG
GTF-*EcoR*I-rev	CCGGAATTCTTAATAATATCTAGCGATACCCC
GTF-418-fwd	ATTCTTCTGGTGGC**NNK**GACTTCCTCCTAGC
GTF-469-rev	AATCTGCAGCAAGTCGGCATTAAC**MNN**ATCAAC
GTF-D-pET-*BamH*I-fwd	CCGGGATCCACAGAACAGCAGACCTCAG
GTF-D-pET-*EcoR*I-rev	CCGGAATTCTTAATAATATCTAGCGATACCCC
M4-N413A-fwd	AATATTTTGAAGAC**GCA**TCTTCTGGTGGCCG
M4-N413A-rev	CGGCCACCAGAAGA**TGC**GTCTTCAAAATATT

Note: restriction sites were indicated by underlines

Table S2 ^1H and ^{13}C NMR data of G1, G2 and C1

**Position**	**δ_CG1**	**δ_HG1**	**δ_CG2**	**δ_HG2**	**δ_CC1**	**δ_HC1**
**2**	154.3 (CH)	8.13 (s)	154.3 (CH)	8.12(s)	82.6 (CH)	4.60 (d, 6.4)
**3**	123.5 (C)		123.5 (C)		68.9 (CH)	4.0 (m)
**4**	181.8 (C)		181.4 (C)		28.6 (CH_2)	a2.80 (dd,4.8,2.4)<br>b2.49 (dd, 7.6,7.6)
**5**	163.5 (C)		164.1 (C)		158.0 (C)	
**6**	101.3 (CH)	6.87 (s)	100.7 (CH)	6.69 (s)	96.4 (CH)	5.91 (s)
**7**	164.2 (C)		166.6 (C)		157.7 (C)	
**8**	95.9 (CH)	6.95 (s)	95.1 (CH)	6.77 (s)	95.6 (CH)	5.84 (s)
**9**	158.5 (C)		159.1 (C)		156.8 (C)	
**10**	107.8 (C)		106.2 (C)		100.8 (C)	
**1'**	122.6 (C)		125.8 (C)		136.4 (C)	
**2'**	131.5 (CH)	7.70 (d, 8.0)	131.2 (CH)	7.68 (d, 8.0)	116.1 (CH)	6.90 (s)
**3'**	116.8 (CH)	7.30 (d, 8.0)	117.8 (CH)	7.48(d, 8.4)	148.7(C)	
**4'**	159.8 (C)		158.9 (C)		146.2 (C)	
**5'**	116.8 (CH)	7.30 (d, 8.0)	117.8 (CH)	7.48 (d, 8.0)	118.8 (CH)	7.23 (d, 7.6)
**6'**	131.5 (CH)	7.70 (d, 8.0)	131.2 (CH)	7.68 (d, 8.0)	120.0 (CH)	6.80 (d, 7.2)
**1"**	99.9 (CH)	6.29 (d,2.4)	100.2 (CH)	6.13 (d,3.2)	102.0 (CH)	5.37 (d,1.6)
**2"**	73.7 (CH)	4.39 (m)	74.0 (CH)	4.39 (m)	71.7 (CH)	3.60 (s)
**3"**	76.5 (CH)	4.46 (m)	75.9 (CH)	4.52 (m)	75.2 (CH)	3.57 (m)
**4"**	72.1 (CH)	4.43 (m)	72.3 (CH)	4.44 (m)	71.2 (CH)	3.59 (m)
**5"**	75.6 (CH)	4.82 (m)	75.8 (CH)	4.88 (m)	72.2 (CH)	3.61 (m)
**6"**	62.9 (CH_2)	4.54 (m)	63.0 (CH_2)	4.46 (m)	62.2 (CH_2)	3.75 (m)

G1: genistein-7-*O*-α-D-glucopyrasoside; G2: genistein-4'-*O*-α-D-glucopyranoside. Spectra were measured at 100 MHz for ^{13}C and 400 MHz for ^1H in pyridine-*d*5.

C1: catechin-4'-*O*-α-D-glucopyranoside.Spectra were measured at 100 MHz for ^{13}C and 400 MHz for ^1H in MeOD.
